# Genetic and phenotypic diversification in a widespread fish, the Sailfin Molly (*Poecilia latipinna*)

**DOI:** 10.1186/s12862-024-02270-x

**Published:** 2024-07-01

**Authors:** Ralph Tiedemann, Rüdiger Riesch, Maxi Tomowski, Katja Havenstein, Jan Schlupp, Waldir Miron Berbel-Filho, Ingo Schlupp

**Affiliations:** 1 Unit of Evolutionary Biology/Systematic Zoology Institute of Biochemistry and Biology, University of Potsdam, Potsdam, Germany; 2 Department of Biological Sciences, Royal Holloway University of London, Egham, TW20 0EX UK; 3 CEFE, CNRS, Univ Montpellier, EPHE, IRD, Montpellier, France; 4 School of Biological Sciences, University of Oklahoma, 730 Van Vleet Oval, Norman, OK 73019 USA; 5 International Stock Center for Livebearing Fishes, University of Oklahoma, Norman, OK 73019 USA; 6 Amazon, amazon.com, Arlington, VA, 22202, USA; 7 Department of Biology, University of West Florida, Pensacola, FL, 32514, USA

**Keywords:** Genetic structure, Palaeodrainages, Life history, Dispersal, Poeciliidae

## Abstract

Widespread species often experience significant environmental clines over the area they naturally occupy. We investigated a widespread livebearing fish, the Sailfin molly (*Poecilia latipinna*) combining genetic, life-history, and environmental data, asking how structured populations are. Sailfin mollies can be found in coastal freshwater and brackish habitats from roughly Tampico, Veracruz in Mexico to Wilmington, North Carolina, in the USA. In addition, they are found inland on the Florida peninsula. Using microsatellite DNA, we genotyped 168 individuals from 18 populations covering most of the natural range of the Sailfin molly. We further determined standard life-history parameters for both males and females for these populations. Finally, we measured biotic and abiotic parameters in the field. We found six distinct genetic clusters based on microsatellite data, with very strong indication of isolation by distance. However, we also found significant numbers of migrants between adjacent populations. Despite genetic structuring we did not find evidence of cryptic speciation. The genetic clusters and the migration patterns do not match paleodrainages. Life histories vary between populations but not in a way that is easy to interpret. We suggest a role of humans in migration in the sailfin molly, for example in the form of a ship channel that connects southern Texas with Louisiana which might be a conduit for fish migration.

**Supplementary Information:**

The online version contains supplementary material available at 10.1186/s12862-024-02270-x.

## Introduction

Patterns of biodiversity and speciation continue to intrigue evolutionary biologists and ecologists [1]. At the nexus of this debate is the question about the mechanisms that lead to differentiation and potentially speciation. Nonetheless, the flipside of this, the lack of differentiation where it may be predicted, is equally important to consider. For example, many organisms are geographically widespread, with multiple populations exhibiting different degrees of reproductive isolation and experiencing various ecological conditions. Yet they appear to exhibit little to no diversification at the species level [2], which could be due to a host of factors, including a lack of geographic isolation amongst populations, intense gene flow, demography, or short time since population establishment [3, 4]. There are examples of genetic differentiation in the absence of phenotypic differentiation, for example in *Bororas maculatus* from Malaysia [5] and in *Microrasbora rubescens* from Myanmar [6]. This is in stark contrast to the rapid differentiation and even speciation found in some taxa, such as cichlid fishes of the East African rift valley [7], *Laupala* crickets in Hawaii [8], and toads [9].

Sometimes, cryptic diversity can be detected at the molecular level, but is not reflected in taxonomically relevant morphological traits. Cryptic diversity in bryophytes is a great example of this [10]. Thus, understanding the patterns of cryptic diversity is crucial for understanding the evolution of biodiversity [11], and additionally may provide guidance for the conservation of species [12]. In fact, cryptically diverse taxonomic units may present different responses to Global Change [13].

One driving factor of cryptic diversity is geographic isolation (i.e., allopatry), which, in freshwater fishes, is usually related to water connectivity [14]. Indeed, freshwater fishes are models to understand the biogeographical history of a region, given that their natural distribution and dispersal relies on both current and historical connections between river drainages. Two major events are frequently invoked to explain the disjunct distribution of freshwater fishes among isolated rivers: (i) river captures, an inland event that happens when a river system changes its course and connects with another drainage system due to geological activities [15] and, (ii) paleo-drainage connections, a coastal event that refers to sea level fluctuations caused by climatic changes, occasionally causing basins that previously were isolated to coalesce (sea level decrease), or previously-connected river basins to become isolated (sea level increase) [16]. Geographical isolation between river systems is expected to restrict gene flow and to enhance population and local differentiation.

In the present paper we explore the biogeographical history as well as the genetic and phenotypic diversity of a widespread species, the Sailfin molly (*Poecilia latipinna*) [17]. The sailfin molly is a livebearing fish of the family Poeciliidae. They show ovoviviparity and have internal fertilization [18] (see Supplementary material for additional information). This freshwater fish exhibits an exceptionally large geographical range, compared to some other livebearing fishes. For example, a close relative, *P. latipunctata* [19–21] is only known from a small area in Northeastern Mexico [22]. *Poecilia latipinna* typically has a narrow coastal distribution that follows the coastline first along the Gulf of Mexico, around the tip of Florida, and then along the Atlantic Coast of the US, covering ca. 4,600 km of coastline. In Florida, it is also widely found inland [23].

Their overall distribution range predicts that Sailfin mollies should be experiencing several ecological clines, which might provide opportunities for local adaptation and restriction of gene flow, which might in turn lead to genotypic and phenotypic divergence. Yet at least some of the morphological variability associated with geographical distance seems to be due to phenotypic plasticity [17]. For example, Sailfin mollies experience high temperatures in the summer of over 35ºC in Tampico, Tamaulipas [24] to winter temperatures below freezing in Wilmington, North Carolina [25]. Ecological niche modeling has indicated that temperature largely determines the distribution of Sailfin mollies, with the lowest winter temperatures defining the northern limit of the range [26]. This is particularly relevant because many aspects of fish life histories are temperature-dependent (e.g., in sailfin mollies: [17]; eastern mosquitofish, *Gambusia holbrooki*: [27]; *Phalloptychus januarius*: [28]; pumpkinseed, *Lepomis gibbosus*: [29]. Such a cline in temperatures may lead to locally adapted populations. On the other hand, Sailfin mollies are also known to be euryhaline occurring in both freshwater and coastal lagoons, and being able to tolerate marine [30, 31] and even hypersaline conditions [32]. This ability may allow them to migrate between river systems, effectively limiting local adaptation and population differentiation via dispersal and gene flow.

Sailfin mollies are considered ecological generalists and feed on detritus, aquatic invertebrates, and food that lands on the water surface [33]. Furthermore, in their food-web, they are prey for fishes, birds, and reptiles [34]. In addition to their native range, they have been introduced widely (e.g., in Central Texas [35, 36]), either because they were used as bait, or through the aquarium trade, where Sailfin mollies are considered both attractive and hardy pet fish [37]. Overall, Sailfin mollies have long been a model for research in behavior, ecology, and evolution [38–46] (for additional background on Sailfin mollies see Supplementary Backgound Information).

Using the Sailfin molly as a model system to address evolutionary questions in widespread species we apply a multifaceted approach to analyze patterns of potential cryptic biodiversity. Based on samples taken along most of the natural range of the Sailfin molly we used microsatellite data to detect cryptic variability and infer population genetic structure. This genetic analysis was then used as a baseline for subsequent analyses of phenotypic traits that should be sensitive to environmental clines, namely life-history traits. Hence, in this study, we were interested in utilizing the power of genetic population delimitation to predict differences in life history.

More precisely we addressed the following questions: (1) Is there cryptic genetic diversity in *P. latipinna*? (2) Does the genetic structuring coincide with the biogeographical history of the region? Finally, (3) is variation in life-history traits linked to the observed genetic variability? We hypothesize that based on the wide distribution of the species there might be local adaptation and cryptic diversity. We also hypothesize that, provided that genetic diversity is governed by biogeographical barriers, life histories will differ between the genetic lineages.

## Materials and methods

### Sampling

Specimens were collected in the summer of 2008 between June 10 and 20. Starting near Angleton, Texas, for the main field campaign, we (RR, JS, IS) collected samples east and north along the coastline of the Gulf of Mexico to Florida. Northern Florida was crisscrossed to additionally sample inland populations, while Southern Florida was not sampled due to logistical limitations. After sampling Florida, we followed the coastline to North Carolina, where the northernmost native population was sampled in Wilmington, North Carolina. We further added a site from Brownsville, South Texas, on July 1, 2008 (RR, IS). *Poecilia latipinna* is also found in Mexico to roughly the mouth of the Rio Tuxpan, but this area was not sampled due to logistical constraints. We assume that our study only includes natural populations. We did - for example - ignore populations in Central Texas which had been introduced in the 1950’s, presumably from Florida [35].

Conditions permitting, we established a sampling site approximately every 50 km. At every sampling site, we measured several habitat variables using a Hydrolab Datasonde 4a Probe with a Surveyor 4 Series Data Display (Hach Company, Loveland, Colorado) (Supplementary Table 1) and took representative habitat photos (not shown). Measuring Chlorophyll a was done with a Turner Designs SCUFA Fluorometer [47].

Fishes were collected with a standard 2 m seine (3.2 mm mesh width) (Memphis Net & Twine, Memphis, Tennessee). We stopped when we had collected at least 20 Sailfin mollies within ca. 30 min of seining or were unable to collect more. Hence, catch per unit effort varied with site. Specimens were sacrificed in the field and immediately stored in 70% ethanol. This way, we sampled a total of 46 sites between Brownsville, TX and Wilmington, NC. We used 18 populations for population genetic analysis and subsequent life-history analysis, with a minimum requirement of five collected specimens per population. Data from the same sampling campaign were previously published in studies on mtDNA [48] and multiple paternity in Sailfin mollies [49].

### Molecular analyses

**Fig. 1:**
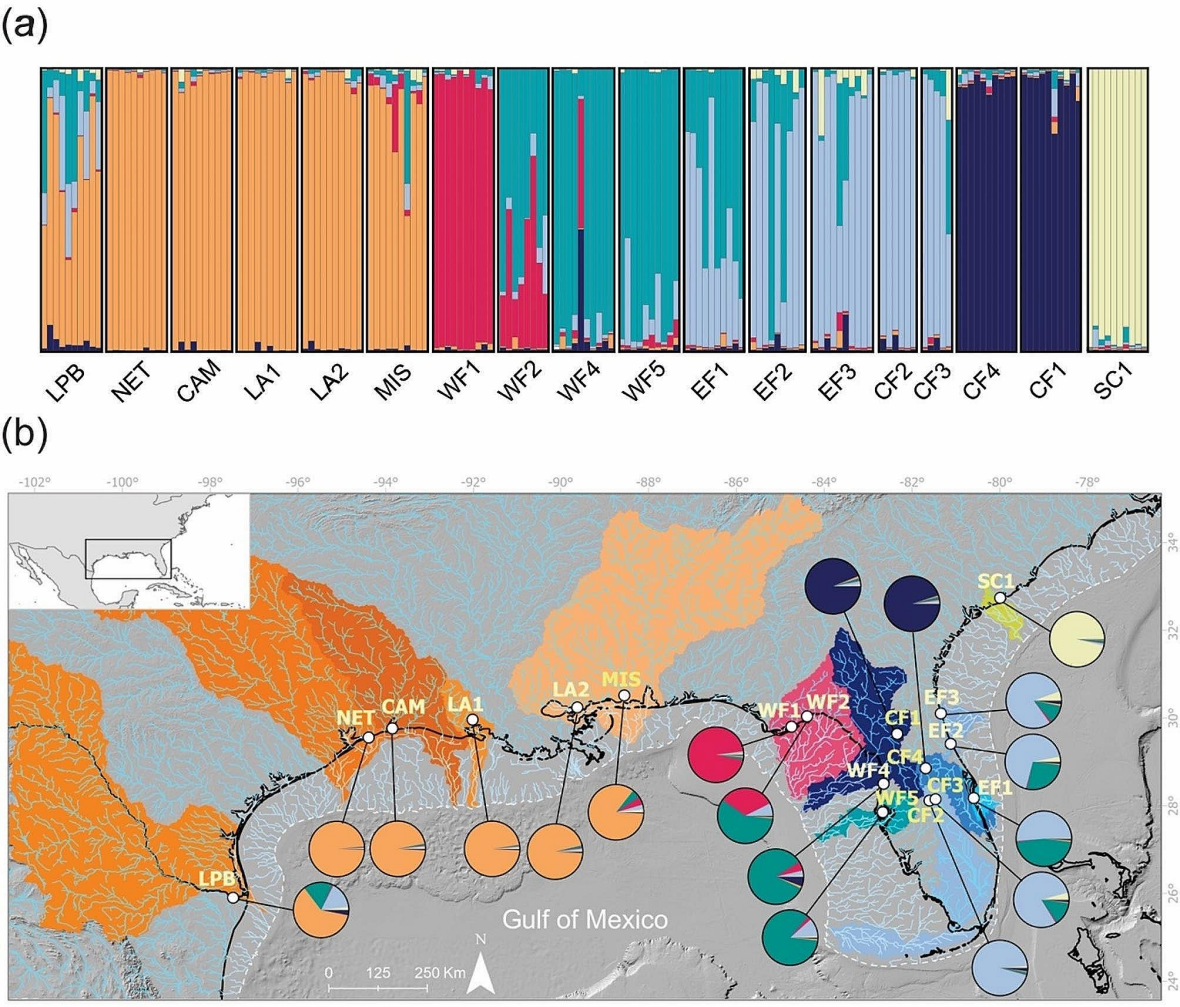
Genetic structure and its spatial distribution of *P. latipinna* genetic clusters. (**a**) Membership-coefficient plot with STRUCTURE results with optimal k = 6 inferred by Bayesian analysis. Each bar represents an individual genotype ordered by sampling location. Distinct colors indicate the relative cluster membership probability. (**b**) Sampling area, palaeodrainage reconstruction, and geographical distribution of genetic clusters found in Sailfin mollies. Pie charts colors represent the average membership proportion for all genetic clusters within a sampling site. Colors in the continent (and palaeodrainages) represent river basins reconstructed via palaeodrainage reconstruction (see Methods below). Dark solid line represents current coastline (0 m above sea level); dashed white line denotes sea level at the last glacial maximum (125 m below current sea level.) Orange cluster: west of Alabama; Red cluster: northwest Florida; Green cluster: Florida west coast; Light-blue cluster: Florida east coast; Dark-blue cluster: Florida inland; Yellow cluster: South Carolina

We sampled dorsal fin clips from 18 populations (*n* = 168) of *Poecilia latipinna* along the Gulf of Mexico and the Atlantic coast of Florida, South and North Carolina (Fig. 1; see Supplementary Table 2 for location details).

DNA was isolated as in Tiedemann (2005). We genotyped 14 microsatellite loci originally developed for a closely related species (*P. formosa*), namely (primer set I) GT-I41, GA-IV42, GA-I29B, GA-III29B, GA-V18, GA-III49A, GA-II41, GA-I47A, GA-II33, and (primer set II) GA-I13B, GA-I5B, GT-I34, GT-I49, GA-I26 [50]. About 100ng of genomic DNA were used as template. PCR was performed in a total volume of 25 µl, containing 10mM Tris-HCl, pH 9.0, 50mM KCl, 1.5mM (primer set I) resp. 3mM (primer set II) MgCl_2_, 0.2mM (primer set I) resp. 1mM (primer set II) of each dNTP, 0.136µM (primer set I) resp. 0.16 µM (primer set II) of both forward and reverse primer (one of them 5’-fluorescence-labelled), and 0.5U *Taq* polymerase (MP Biomedicals; primer set I) resp. 0.6U My*Taq* polymerase (Bioline; primer set II). Amplifications were performed in a Biometra TGradient thermocycler according to the following reaction profile: Primer set I: one cycle 94 °C 5 min,18,040 cycles 94 °C 30s, the locus-specific annealing temperature (Supplementary Table 1) 1 min,18,172 °C 45s; a final extension at 72 °C for 10 min. Primer set II: one cycle 95 °C 1 min, 35 cycles 95 °C 15s, the locus-specific annealing temperature (Supplementary Table 1) 15s, 72 °C 10s; a final extension at 72 °C for 10 min. Fragment size was determined on an ABI 3130xl automatic sequencer, using the GENEMAPPER 4.0 software and an internal size standard (GeneScan500(-250)-LIZ, Applied Biosystems). Based on the microsatellite data (Supplementary Table 2) population specific genetic diversity parameters, observed and expected heterozygosity (HO, HE) as well as rarefied allelic richness (AR), total allele number and inbreeding coefficient (FIS), were obtained using the Hierfstat package [47] in R (Version 4.2.2) (Supplementary Table 3). Population specific genetic diversity parameters, observed and expected heterozygosity (H_O_, H_E_) as well as rarefied allelic richness (A_R_), total allele number and inbreeding coefficient (F_IS_), were obtained using the Hierfstat package [51] in R (Version 4.2.2) (Supplementary Table 4). Significant deviation from Hardy-Weinberg-Equilibrium was determined using the hw.test function implemented in the {pegas} package [52] with 1,000 permutations.

We further investigated levels of population differentiation by calculating pairwise *Fst* and *G’st* distances [53, 54] between local populations as well as their respective 95% confidence intervals using 10,000 bootstrap replicates in the {diveRsity} package [55].

To test for a correlation between pairwise genetic distance and geographical Euclidean distance, a Mantel test for isolation by distance (IBD) was performed in R using the {adegenet} package [56]. The significance of the correlation was evaluated using the mantel.randtest function with 10,000 permutations. We conducted a further IBD test based on a reduced sample set omitting populations from central and east Florida to exclude a potentially confounding effect of continental barriers on the IBD inference.

Population structure was inferred using the Bayesian clustering analysis implemented in *STRUCTURE* Version 2.3.4 [57]. We conducted 10 simulations for each putative genetic cluster value (K) from two to 20 with a 50k burn-in period followed by 200k Markov Chain Monte Carlo (MCMC) repetitions under the admixture model without prior population information. The optimal number of clusters was determined by plotting the likelihood of K for each value of K (*LnPr*⌈*X*|*K*⌉) using the △*K* method [58] in the {Pophelper} package [59] in R. Clustering results of individual membership probabilities were visualized with the implemented {pophelperShiny} App.

We conducted a hierarchical AMOVA to estimate variance components among genetic clusters revealed by STRUCTURE and among local populations nested within clusters in Arlequin Version 3.5 [60] using 20,000 permutations.

To identify potential barriers to gene flow, a spatial principal components analysis (sPCA) was performed based on Moran’s I index of spatial autocorrelation in {adegenet}, accounting for differences in allele frequencies between sites depending on geographic distance. We chose the Delaunay triangulation method to weigh network connectivity. Monte Carlo resampling tests with 10,000 iterations were performed to test the statistical significance of global and local spatial structures. The scores of the resulting first eigenvectors of the sPCA were mapped to their corresponding geographic coordinates.

Recent dispersal was assessed by first-generation migrant detection in GENECLASS2 [61]. We applied the Bayesian criterion of [62] and the resampling method proposed by [63] with 10,000 simulations to compute the probability of an individual belonging to its local or an external gene pool using a probability threshold of 0.05.

### Palaeodrainage reconstruction

Palaeodrainage configuration during the Last Glacial Maximum (LGM) was reconstructed using topographic and bathymetric data available in the General Bathymetric Chart of the Oceans website (https://www.gebco.net/). A bathymetric 30 arc-second grid layer containing the Gulf of Mexico area and the United States of America East Coast was uploaded and processed into ArcGIS Pro software, with the Hydrological tools add-in used to reconstruct the palaeodrainages, according to the protocol described in [64]. Briefly, the area exposed during the LGM (-125 m from current sea level) was identified using the tool Contour followed by Mask. The options Fill (to fill the depressions on the surface), followed by Flow Direction (identify the steepness within each cell), Basin (delimit watershed borders), and Stream order (to estimate putative palaeorivers) were used to reconstruct and visualize the palaeodrainages. A detailed protocol for palaeodrainages reconstruction is available at https://github.com/waldirmbf/Paleodrainages.

### Life-history measurements and analyses

Life-history traits could be quantified in 14 populations for males (i.e., NET, CAM, LA1, LA2, MIS, WF1, WF5, CF1, CF3, CF4, EF2, EF3, SC1, and LPB) and 11 populations for females (i.e., CAM, LA1, MIS, WF1, WF5, CF1, CF3, CF4, EF3 and LPB) – based on the availability of sexually mature males and gravid females. Preserved specimens (different individuals than the ones used for population genetics) were sexed based on external characteristics and dissected following well-established protocols [65, 66]. In short, reproductive tissues (testes for males and ovaries for females) were removed and the fish were weighed and measured for standard length. Developing offspring, if present, were separated, counted, and their stage of development determined [67]. All tissues were then dried for 24 h at 55ºC and subsequently weighed again. To assess the amount of soluble fats, tissues were then rinsed at least six times for a minimum of 6 h in petroleum ether [68]. All tissues were reweighed one last time after fat extraction. This protocol provided us with the following four life-history traits for males: SL [mm], lean mass [mg], fat content [%] and gonadosomatic index, GSI [%; male testes mass divided by the sum of male testes dry mass and male somatic dry mass]. For females, we collected the following seven life-history traits: SL [mm], lean mass [mg], fat content [%], fecundity [the number of developing embryos], offspring lean mass [mg], embryo fat content [%] and reproductive allocation, RA [%; total embryo dry mass divided by the sum of total embryo dry mass and female somatic dry mass].

To facilitate meeting model assumptions of most subsequent analyses, life-history data were log_10_-transformed (SL, lean mass, embryo dry and lean mass), square root-transformed (fecundity) or arcsine-transformed (fat content, embryo fat content, GSI, and RA), and then z-transformed (to remove scaling effects). Male size distributions in natural populations of Poeciliids are often bimodal [69, 70]. We therefore first evaluated the distribution of male SL in each population by conducting a series of Shapiro-Wilk tests.

We then ran several preliminary analyses to explore population-level variation in male and female life-history traits. First, we ran two sex-specific univariate general linear models (GLMs) on male and female SL with ‘Genetic Cluster’ (based on *k* identified by STRUCTURE; see above) and ‘Population-nested-within-Genetic Cluster’ [henceforth Population(Genetic Cluster)] as independent variables. This was followed by two sex-specific multivariate GLMs. For males, the dependent variables were lean mass, fat content and GSI, male SL was included as a covariate, and ‘Genetic Cluster’ and ‘Population(Genetic Cluster)’ served as independent variables. For females, the dependent variables were lean mass, fat content, fecundity, embryo lean mass, embryo fat content and RA. As covariates we included female SL and embryonic stage of development and as independent variables, we again used ‘Genetic Cluster’ and ‘Population(Genetic Cluster)’. These preliminary analyses confirmed significant population-level variation (see Supplementary Results), so we proceeded with our planned analyses.

To evaluate if some of the population-specific variation in life-history traits could be explained by local habitat variables, we reduced the variables water temperature, pH, turbidity, Dissolved Oxygen (DO) [%] and salinity via sex-specific Principal Component Analysis – since we had different numbers of populations for analyses of male and female life-history traits. For males, this resulted in two principal components (PCs) being extracted that explained 72.06% of the total variance. PC1 mainly corresponded to water temperature, DO, and salinity, while PC2 mainly corresponded to pH, turbidity and to a lesser extent also DO (Supplementary Table 5). For females, we extracted two PCs with a cumulative variance of 74.38%. Here, PC1 mainly loaded highly for water temperature, DO, salinity, and to a lesser extent also pH, while PC2 mainly represented turbidity and to a lesser extent pH and DO (Supplementary Table 6).

We then used these PCs as covariates in a set of GLMs on male and female life-history traits of the same structure as described above, but the independent variable ‘Population(Genetic Cluster)’ was now replaced by PC1 and PC2 as covariates. All these life-history analyses were conducted using IBM SPSS 28.0.1.1. (IBM Corporation).

Finally, we wanted to evaluate if phenotypic differentiation was associated with genetic differentiation (measured via pairwise *F*_ST_). To that end, we condensed male and female life-history traits via sex-specific PCA, which resulted in 4 PCs capturing 100% of the total variance for males and 6 PCs capturing 100% of the total variance for females. Using population means for each PC and sex, we then calculated pairwise Euclidian distances in PC-space for each sex and correlated them to pairwise *F*_ST_ distances via Mantel tests. Mantel tests were computed using the {permute} library (Simpson, 2022) and the {vegan} library [71] in R version 4.2.1 (R Core Team 2022) with 10,000 permutations.

Raw data is available on Dryad (DOI: 10.5061/dryad.2jm63xsxb) and as supplementary data attached to this manuscript.

## Results

### Population genetics

*Detected clusters*: One microsatellite locus (GA-I26) was monomorphic in all analyzed specimens and hence excluded from further analyses. The remaining 13 loci were all polymorphic with 4 (GT-I13B; GA-I5B) to 42 (GT-I34) alleles (Supplementary Table 3). All analyzed populations were in Hardy Weinberg Equilibrium at all analyzed loci (see Supplementary Tables 3, 4).

Applying the hypothesis-free STRUCTURE approach to delimitate clusters of genetically related individuals detected profound population structure and yielded highest support (δ*K*) for k = 6 clusters (Fig. 1, Supplementary Fig. 1). These clusters were (1) populations west of Alabama, (2) a population adjacent to Alabama in the east (WF1), (3) populations along the west coast of Florida, (4) populations along the east coast of Florida (including two Florida inland populations), (5) inland populations of northern Florida, and (6) South Carolina. Most individuals were assigned to their respective clusters with high probabilities, only the individuals of EF1 and EF2 were ambiguous in their assignment, being mostly intermediate between the two adjacent clusters (Green and light-Blue). In the westernmost sampled population (LPB), we also observed some ambiguity in the assignment which may indicate an influx from further (unsampled) populations along the coast of Mexico. Comparing STRUCTURE assignment to geographic location (Fig. 1) does not only visualize shifts in genetic population structure but indicates some (albeit limited) genetic mixing from (1) WF1 (Red cluster) to adjacent populations to the east and (2) from South Carolina (SC1; Green cluster) to the east coast of Florida. This interpretation is supported by the elevated genetic diversity in these populations (Supplementary Table 4). Running STRUCTURE with higher *k*s (up to 10) tentatively sets apart four further clusters, i.e., LPB, MIS, CF2, and CF1/CF4 (Supplementary Fig. 2).

If we assign populations to groups according to their prevalent STRUCTURE assignment (k = 6), an analysis of molecular variance (AMOVA) apportions significant amounts of 16.9% of the variation to divergence among these groups (F_CT_=0.169; *p* < 0.001) and 9.08% to divergence among populations within groups (F_SC_=0.109; *p* < 0.001), while 74.03% of the variation is found within populations (Table 1).

**Table 1:** AMOVA showing the partition of genetic variation. Groups consist of populations pooled according to STRUCTURE assignment. Significance level is based on 20,000 permutations

Source of variation	Df	Sum of squares	Variance components	% variation	Fixation indices	*p*
Among groups	5	268.889	0.812	16.90	FCT = 0.169	< 0.001
Among populations within groups	12	138.482	0.436	9.08	FSC = 0.109	< 0.001
Within populations	318	1129.801	355.283	74.03		
Total	335	1537.173	479.948		FST = 0.259	< 0.001

Genetic diversity (both heterozygosity and allelic richness) was generally lower in populations from the Gulf of Mexico (Orange cluster west of Alabama; except for the diverse population in Brownsville, TX (LPB)) and at the Atlantic Coast of South Carolina (Yellow cluster), while diversity was considerably higher around Florida, both at coastal and inland habitats (Fig. 1). At several loci, we found shifts in allele sizes between the more western populations (Orange cluster) and the rest. Two loci exhibited a specific allele (GA-I5B) or increased variability (GT-II33) in the populations directly east of Alabama (Red cluster, i.e., WF1 and WF2).

**Fig. 2:**
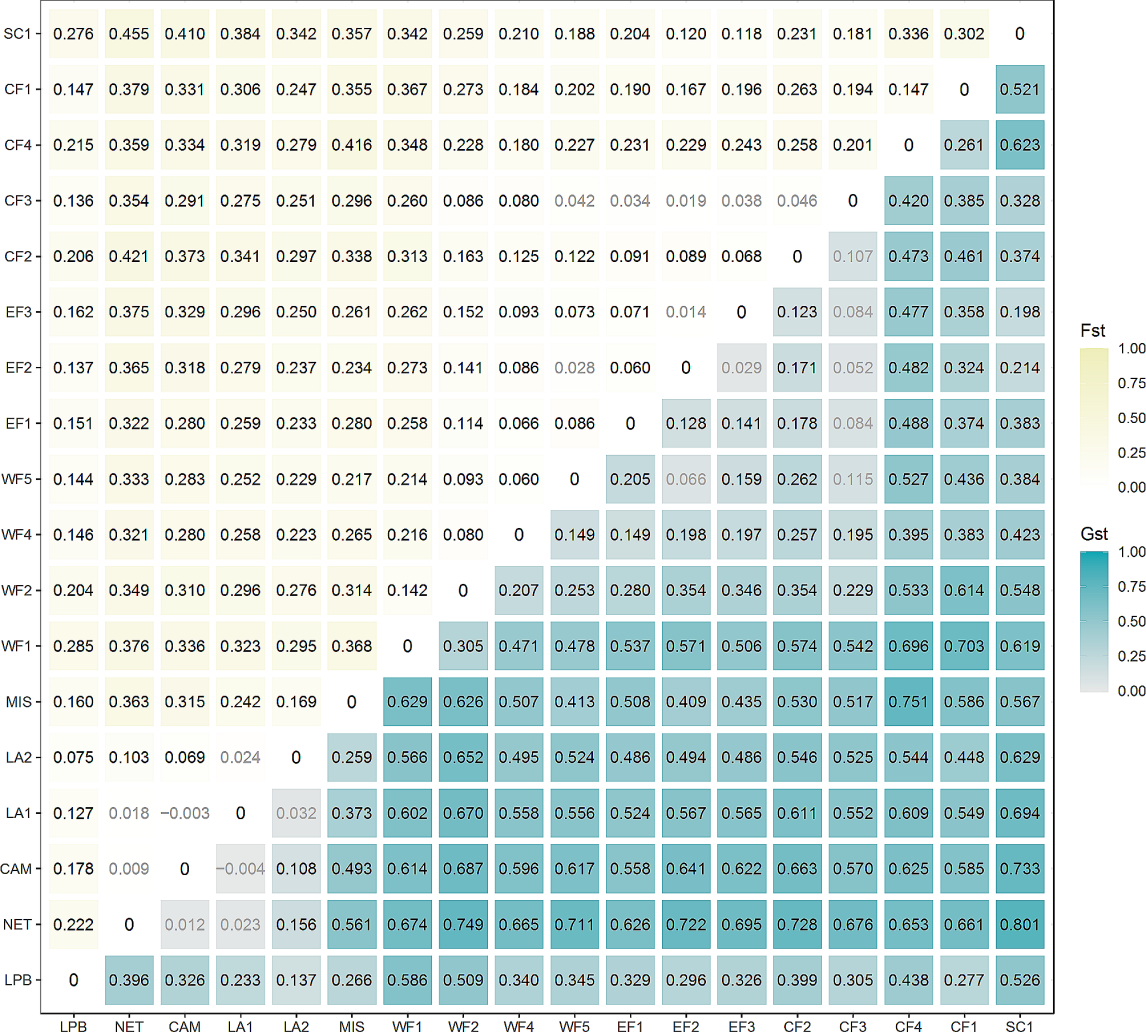
Heatmap and table of pairwise Fst (above diagonal) and G’st (i.e., Fst adjusted for different within populations’ heterozygosity, Hedrick 2005; below diagonal) between *P. latipinna* sampling points; grey (faint) values indicate non-significant G’st values (α = 0.05)

In pairwise comparisons, most populations were significantly diverged from one another (by means of significant fixation coefficient G’_st_ and F_st_, respectively (Fig. 2)). Particularly high divergence among geographically adjacent populations was found west and east of Alabama (i.e., between MIS and WF1, G’_st_=0.628) and between South Carolina (Yellow cluster) and the geographically closest populations of Central Florida (Blue cluster, G’_st_=0.623). There was little or no genetic differentiation among most populations west of Alabama (Orange cluster; NET, CAM, LA1, LA2). Furthermore, one Central Florida population (CF3) was not genetically differentiated from proximate populations on both the east and the west coast of Florida.

**Fig. 3:**
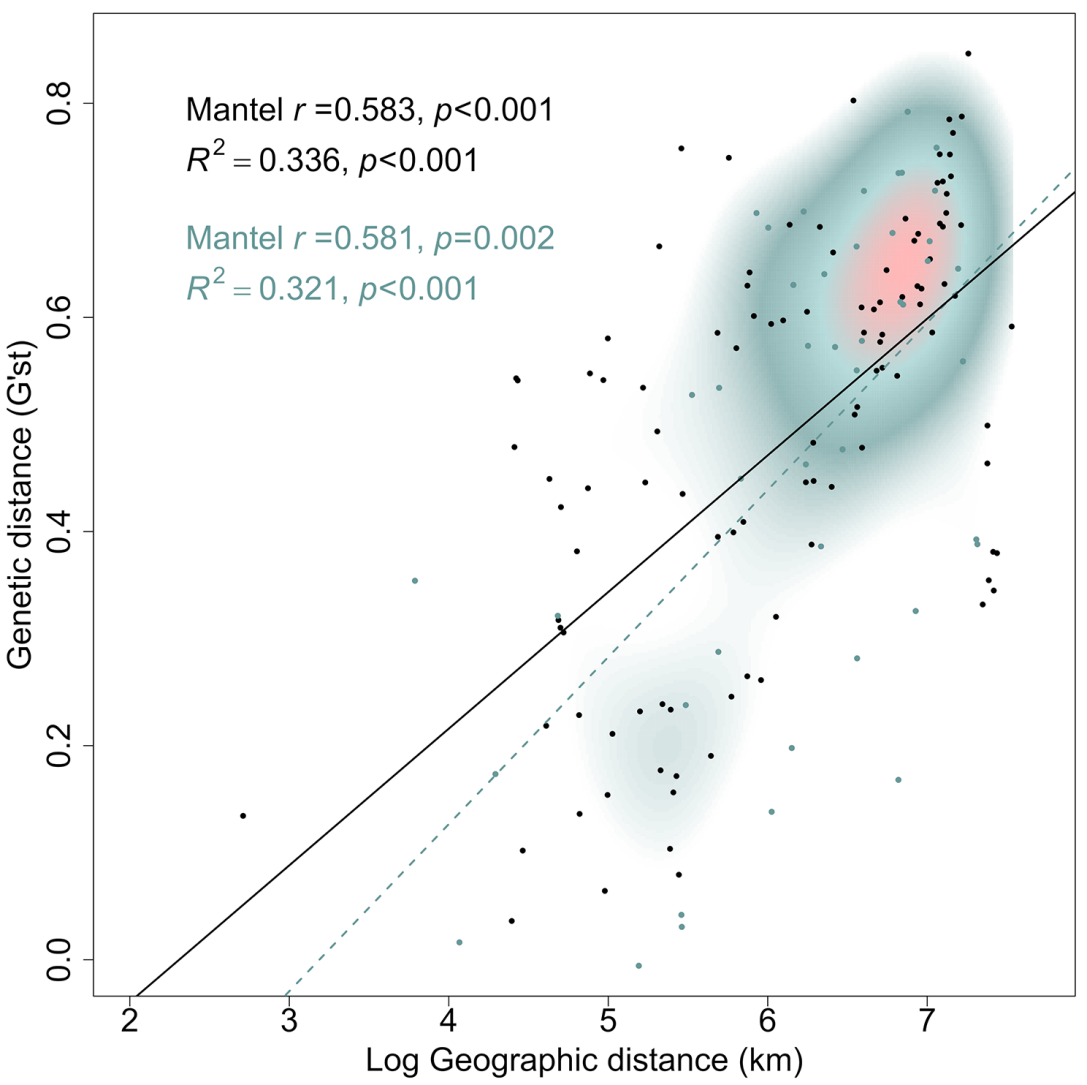
Relationship of (log-transformed) geographic distance and genetic distance. Mantel test statistics are given for both the full data set (black) as well as for a reduced set excluding populations from central and eastern Florida (green)

**Fig. 4:**
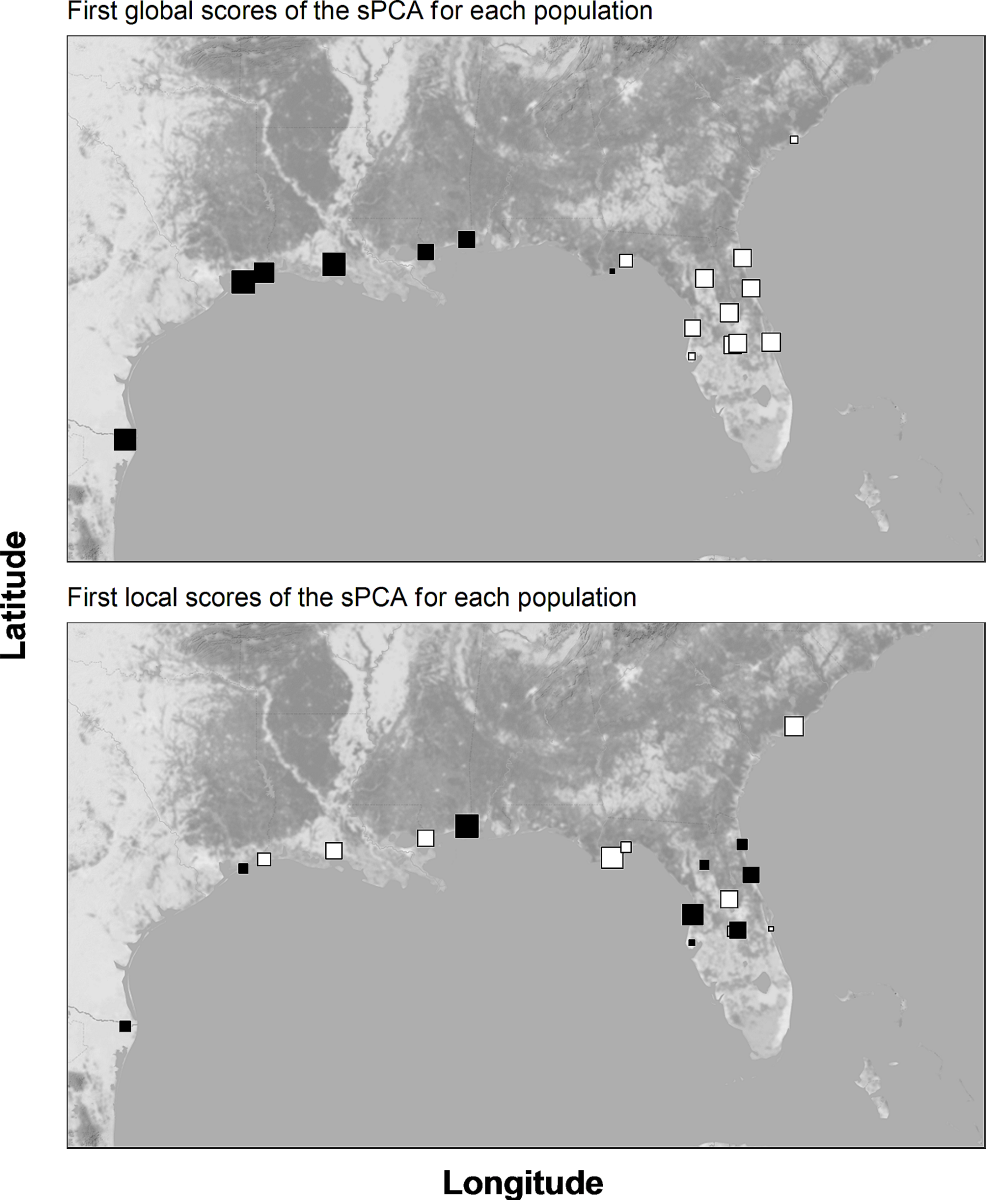
Map showing population scores of the first global and the first local principal component of the spatial principal component analysis (sPCA). Black squares denote negative values, white squares indicate positive values, with size proportional to absolute score

Comparing geographic and genetic (G’st) distance over all pairs of populations by a Mantel test shows highly significant Isolation-by-Distance (*r* = 0.583; *p* < 0.001), accounting for 46.7% of the genetic variation found (Fig. 3). A similar IBD-pattern was observed in the reduced sample that excluded populations from central and eastern Florida to account for continental barriers in IBD inference (see methods, *r* = 0.581; *p* = 0.002). The sPCA revealed a significant global structure, suggesting a correlation between genetic variation and geographic configuration of populations (*p* < 0.001). Accordingly, the mapping of scores of the first principal component illustrates a genetic cline from west to east (Fig. 4). However, tests evaluating local spatial structure showed no significance (*p* = 0.999). In general, the isolation-by-distance results suggest a low capacity for long-distance dispersal in *P. latipinna*.

*Geographical distribution of clusters and paleodrainages*: Our palaeodrainage reconstruction revealed 13 palaeoriver systems in which our 18 sampling sites with genetic data were located (Fig. 1b). Plotting the average genetic clusters by sampling site revealed an interesting pattern: most of the genetic clusters followed the topology of multiple palaeoriver systems. For instance, the Orange cluster (west of Alabama) was distributed across four different paleoriver systems. In West Florida, two major genetic clusters (Red and Green) spanned across three different paleoriver systems. In East Florida and parts of Central Florida, the light blue lineage predominated, however, with a substantial level of admixture with the Green cluster from East Florida, being particularly admixed in the more coastal sampling sites. Still in Central Florida, the dark-Blue cluster was dominant and shared among two populations (CF1 and CF4), which belong to two different palaeodrainage systems, suggesting fish movement between sites. Finally, the Yellow cluster is dominant in a single palaeodranaige system in coastal South Carolina. In general, the spatial distribution of genetic lineages seemed to not be limited by the water boundaries of their paleorivers, with multiple instances of genetic clusters spanning several paleodrainages, as well as multiple paleorivers containing more than one genetic cluster.

**Fig. 5:**
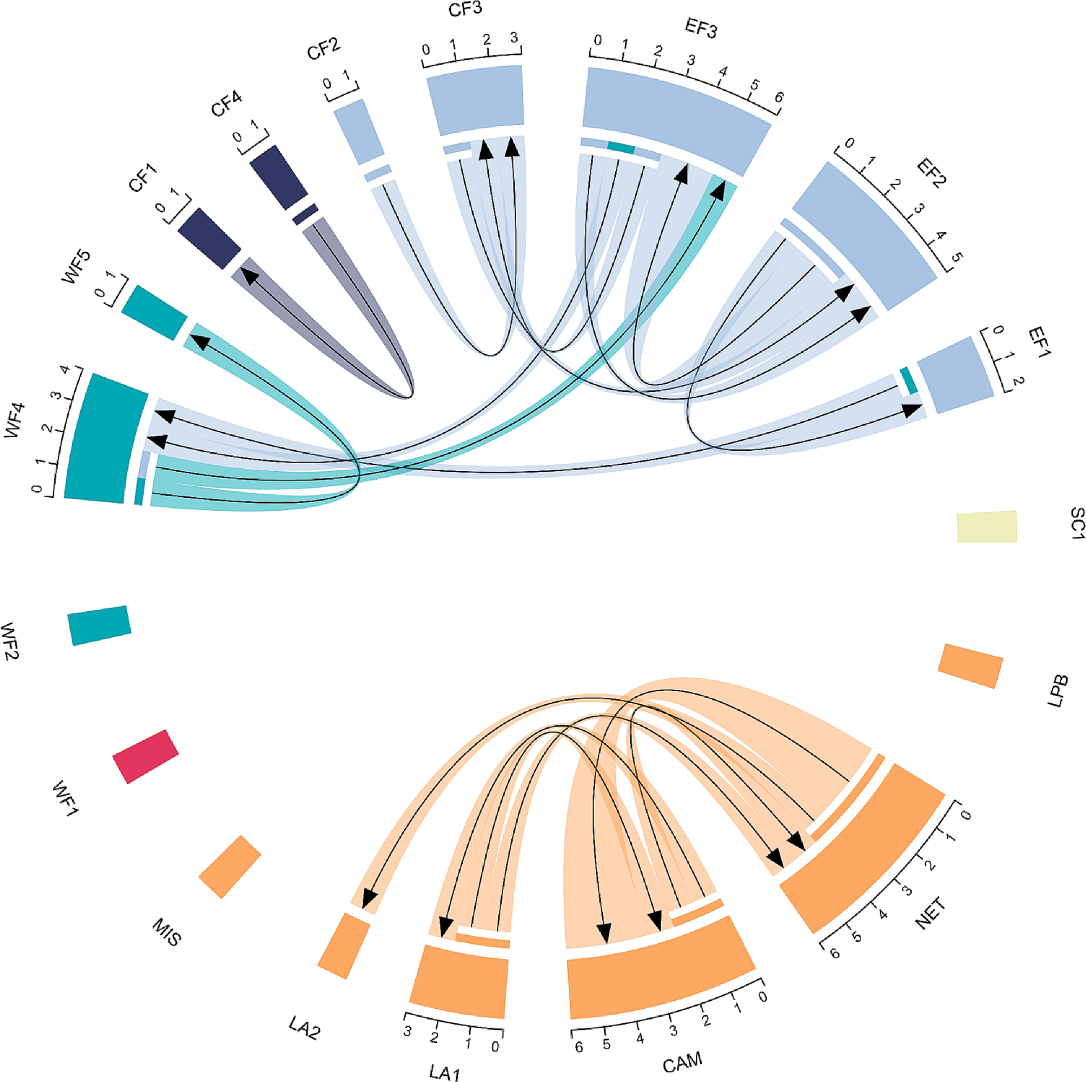
Migration analysis Chord diagram showing first-generation migrants of *Poecilia latipinna* inferred by GENECLASS2 between sampling sites, with scaled sectors showing the sum of immigrants and emigrants detected, the link root/end width proportional to the number of migrants and arrows illustrating the direction of migration. Colors indicate the genetic clusters identified by STRUCTURE analysis

*Migration events*: 20 individuals (11.9%) were identified as first-generation migrants using the L_home_ method in GENECLASS2 (Fig. 5). While most migration events occurred within the same genetic cluster, the majority of them (16/20, Fig. 5) occur between sampling localities within different paleoriver systems. We detected four migration events across cluster borders involving locations of the west- and east coast of Florida (EF3, EF1, WF4). Overall, inferred recent dispersal occurred over medium to longer distances reaching several tens, and in rare cases, a few hundred kilometers.

### Life-history patterns

**Fig. 6:**
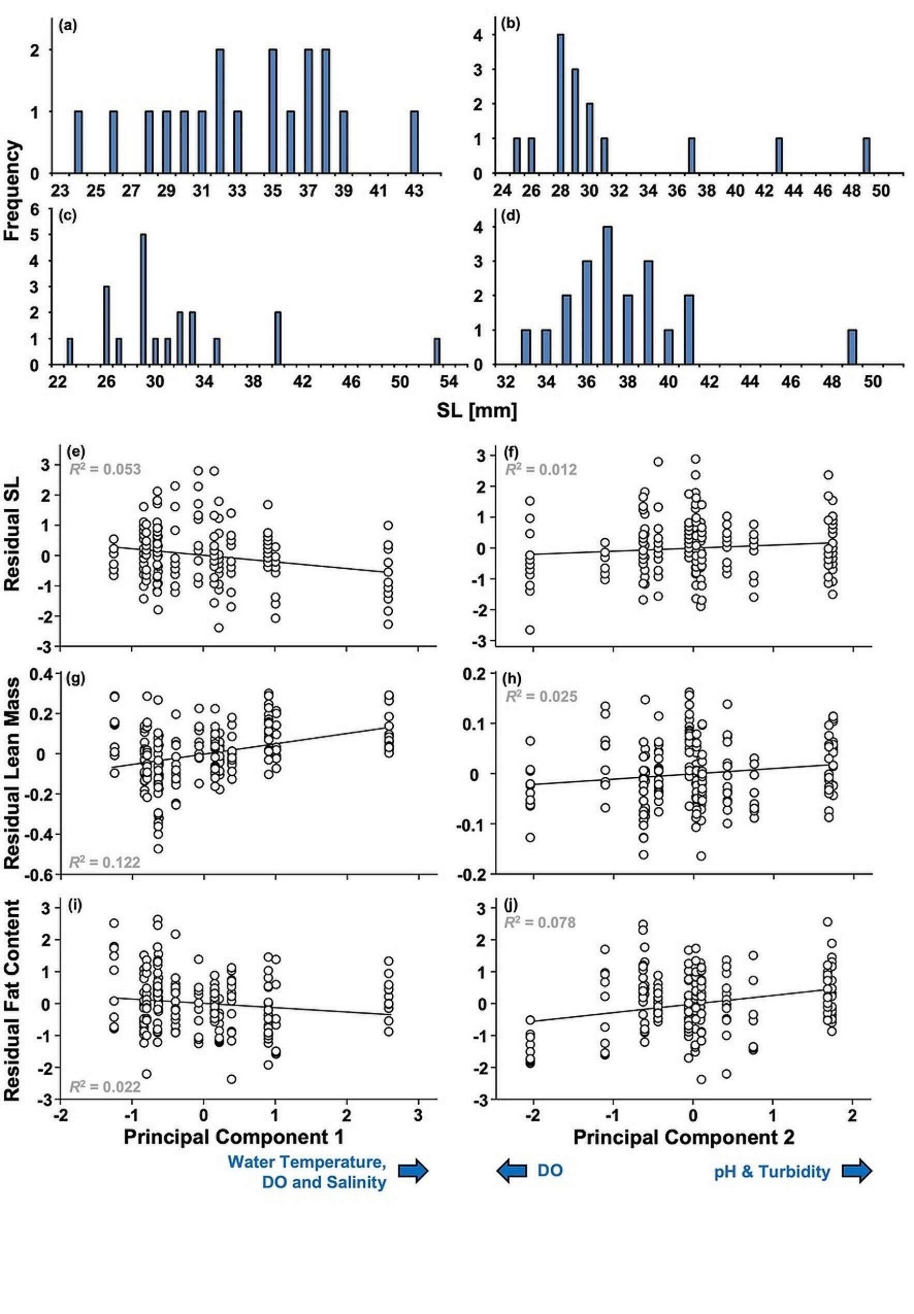
Patterns of male standard length (SL) for four different populations (**a**-**d**). Male size did not deviate from normality in most populations (NET as an example in a) but was not normally distributed in the three sites CAM, CF4, and EF2 (**b**-**d**). Significant effects of our environmental Principal Components on male life histories (**e**-**j**). Male standard length (SL, e-f) and male fat content (**i**-**j**) are corrected for the significant influence of the respective other Principal Component (**e**-**f**), while lean mass (**g**-**h**) is corrected for differences in male SL and the respective other Principal Component

Male size was normally distributed in 11 out of 14 populations (*P* ≥ 0.255 in all cases) but deviated from normality in CAM (*W*_15_ = 0.735, *P* < 0.001), CF4 (*W*_20_ = 0.824, *P* = 0.002), and EF2 (*W*_20_ = 0.854, *P* = 0.006). However, we did not detect bimodality in any of these three populations; rather, distributions were right-skewed with a few very large males (Fig. 6).

**Fig. 7:**
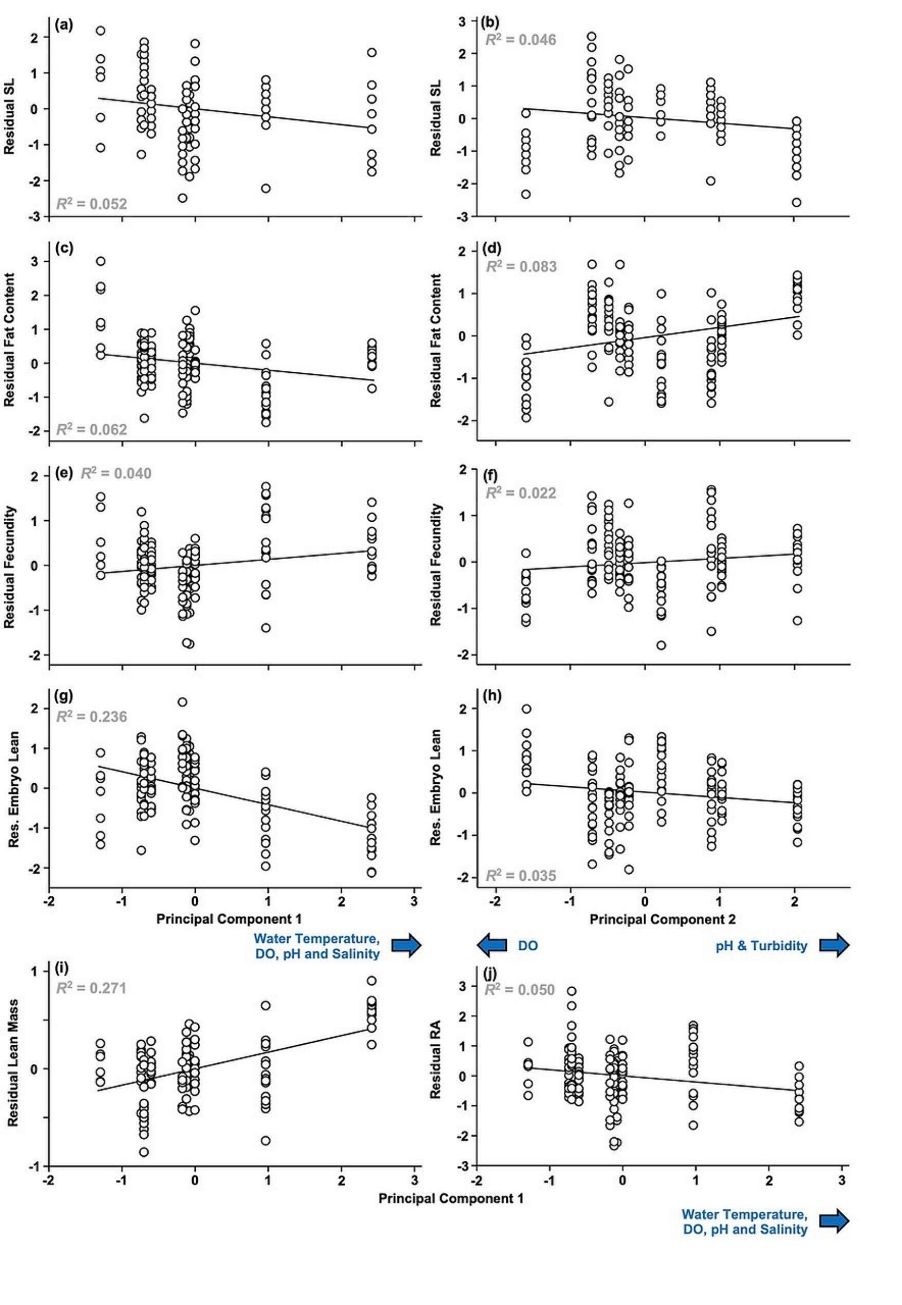
Significant effects of our environmental Principal Components on female life histories. Female standard length (SL; **a**-**b**), fat content (**c**-**d**) fecundity (**e**-**f**) and embryo lean mass (**g**-**h**) are corrected for the significant influence of the respective other Principal Component; fecundity (**e**-**f**), lean mass (**i**) and reproductive allocation (RA; j) are also corrected for differences in female SL, while RA is further corrected for the significant effect of embryonic stage of development (**j**)

The environmental GLM on male SL revealed significant effects of both PCs [PC1: *F*_1,187_ = 19.306, *P* < 0.001, *η*_p_^2^ = 0.094; PC2: *F*_1,187_ = 4.093, *P* = 0.044, *η*_p_^2^ = 0.021] and of Genetic Cluster (*F*_5,187_ = 10.661, *P* < 0.001, *η*_p_^2^ = 0.222). Along PC1, the relationship was negative, so males were smaller in habitats with higher water temperatures, more dissolved oxygen, and greater salinity, while the relationship with PC2 was positive indicating males were larger in habitats with greater pH and turbidity. Regarding genetic clusters, males from Cluster 4 were the largest, Cluster 3 males were the smallest, and males from the remaining four clusters were intermediate. For female SL the results were similar (PC1: *F*_1,131_ = 9.339, *P* = 0.003, *η*_p_^2^ = 0.067; PC2: *F*_1,131_ = 13.617, *P* < 0.001, *η*_p_^2^ = 0.094; Genetic Cluster: *F*_4,131_ = 3.680, *P* = 0.007, *η*_p_^2^ = 0.101). While Cluster 3 had both the smallest males and females, SL was largest for females from Cluster 5, with the remaining Clusters intermediate. Visual examination revealed negative relationships between female SL and both PCs, so that females got smaller with increasing water temperature, dissolved oxygen, salinity, turbidity, and pH (Fig. 7, Supplementary Table 7).

### Males

The multivariate environmental GLM on the remaining male traits returned significant effects of the covariate SL (*F*_3,184_ = 3167.583, *P* < 0.001), both PCs (PC1: *F*_3,184_ = 24.618, *P* < 0.001; PC2: *F*_3,184_ = 12.962, *P* < 0.001) and Genetic Cluster (*F*_15,508_ = 6.419, *P* < 0.001). However, compared to SL (*η*_p_^2^ = 0.981), the effects of PC1 (0.286), PC2 (0.174) and Genetic Cluster (0.148) were much weaker. Post-hoc univariate comparisons (Bonferroni-corrected significance at ⍺ = 0.017) demonstrated that SL significantly affected male lean mass (*F*_1,186_ = 9427.448, *P* < 0.001, *η*_p_^2^ = 0.981) and GSI (*F*_1,186_ = 38.029, *P* < 0.001, *η*_p_^2^ = 0.170), but not fat content (*F*_1,186_ = 2.742, *P* = 0.099, *η*_p_^2^ = 0.015). While male lean mass increased with increasing SL, GSI decreased with increasing SL so that larger males had smaller relative testis mass. Genetic Clusters significantly differed in all three male traits (lean mass: *F*_5,186_ = 3.362, *P* = 0.006, *η*_p_^2^ = 0.083; fat content: *F*_5,186_ = 10.299, *P* < 0.001, *η*_p_^2^ = 0.217; GSI: *F*_5,186_ = 4.680, *P* < 0.001, *η*_p_^2^ = 0.112; Supplementary Table 5). Male lean mass was greatest in Clusters 3 and 6 and by far smallest in Cluster 2, while fat content was highest in Cluster 2 and lowest in Cluster 5, and GSI was greatest in Cluster 6 and lowest in Clusters 2, 4 and 5. Both PCs significantly affected lean mass (PC1: *F*_1,186_ = 57.894, *P* < 0.001, *η*_p_^2^ = 0.237; PC2: *F*_1,186_ = 9.051, *P* = 0.003, *η*_p_^2^ = 0.046) and fat content (PC1: *F*_1,186_ = 8.453, *P* = 0.004, *η*_p_^2^ = 0.043; PC2: *F*_1,186_ = 30.947, *P* < 0.001, *η*_p_^2^ = 0.143) but not GSI (PC1: *F*_1,186_ = 1.437, *P* = 0.232, *η*_p_^2^ = 0.008; PC2: *F*_1,186_ = 0.182, *P* = 0.670, *η*_p_^2^ = 0.001). Visual examination revealed that lean mass increased with increasing temperature, oxygen content, and salinity (PC1), but also with increasing turbidity (PC2), while fat content decreased with increasing temperature, oxygen content, and salinity (PC1), but increased with increasing turbidity (PC2; Fig. 6a-d).

### Females

The multivariate environmental GLM on the remaining female life-history traits indicated significant effects of the covariates SL (*F*_6,124_ = 375.528, *P* < 0.001) and Embryonic Stage of Development (*F*_6,124_ = 9.585, *P* < 0.001), both PCs (PC1: *F*_6,124_ = 47.889, *P* < 0.001; PC2: *F*_6,124_ = 12.941, *P* < 0.001) and Genetic Cluster (*F*_24,434_ = 8.904, *P* < 0.001). The strongest effects were due to SL (*η*_p_^2^ = 0.948) and PC1 (0.699), while the effects of Embryonic Stage of Development (0.317), PC2 (0.385) and Genetic Cluster (0.295) were much weaker. Post-hoc univariate comparisons (Bonferroni-corrected significance at ⍺ = 0.008) demonstrated that SL significantly affected female lean mass (*F*_1,129_ = 1676.468, *P* < 0.001, *η*_p_^2^ = 0.929), fecundity (*F*_1,129_ = 172.164, *P* < 0.001, *η*_p_^2^ = 0.572), and RA (*F*_1,129_ = 11.007, *P* = 0.001, *η*_p_^2^ = 0.079; *P* > 0.104 for all other traits), with all three increasing with increasing female SL (Supplementary Fig. 3, a and b). Embryonic Stage of Development, on the other hand, significantly affected embryo fat content (*F*_1,129_ = 39.057, *P* < 0.001, *η*_p_^2^ = 0.232) and RA (*F*_1,129_ = 17.212, *P* < 0.001, *η*_p_^2^ = 0.118; *P* > 0.032 for all other traits), with both traits decreasing with increasing developmental stage (Supplementary Fig. 3, c and d). Genetic Cluster significantly affected all traits (female lean mass: *F*_4,129_ = 4.716, *P* = 0.001, *η*_p_^2^ = 0.128; female fat content: *F*_4,129_ = 19.521, *P* < 0.001, *η*_p_^2^ = 0.377; fecundity: *F*_4,129_ = 9.300, *P* < 0.001, *η*_p_^2^ = 0.224; embryo fat content: *F*_4,129_ = 3.758, *P* = 0.006, *η*_p_^2^ = 0.104; embryo lean mass: *F*_4,129_ = 8.976, *P* < 0.001, *η*_p_^2^ = 0.218; RA: *F*_4,129_ = 4.982, *P* < 0.001, *η*_p_^2^ = 0.133). Female lean mass was greatest in Genetic Clusters 1 and 5, intermediate in Cluster 4 and smallest in Clusters 2 and 3, while female fat content was highest in Genetic Cluster 2 and lowest in Genetic Cluster 5 (Supplementary Table 6). Fecundity was greatest in Cluster 1 and by far the lowest in Cluster 5, and the opposite pattern was discovered for embryo lean mass, which was by far the greatest in Cluster 5 and lowest in Cluster 1. Embryo fat content was greatest in Clusters 3 and 5 and lowest in Cluster 1, and RA was by far lowest in Cluster 5 and greatest in Clusters 2 and 3 (Supplementary Table 6). PC1 significantly affected all traits but embryo fat content (female lean mass: *F*_1,129_ = 78.952, *P* < 0.001, *η*_p_^2^ = 0.380; female fat content: *F*_1,129_ = 12.311, *P* < 0.001, *η*_p_^2^ = 0.087; fecundity: *F*_1,129_ = 7.629, *P* = 0.007, *η*_p_^2^ = 0.056; embryo lean mass: *F*_1,129_ = 63.764, *P* < 0.001, *η*_p_^2^ = 0.331; RA: *F*_1,129_ = 9.664, *P* = 0.002, *η*_p_^2^ = 0.070; embryo fat content: *F*_1,129_ = 0.585, *P* = 0.446, *η*_p_^2^ = 0.005) and PC2 significantly affected female fat content (*F*_1,129_ = 43.108, *P* < 0.001, *η*_p_^2^ = 0.250), fecundity (*F*_1,129_ = 9.009, *P* = 0.003, *η*_p_^2^ = 0.065) and embryo lean mass (*F*_1,129_ = 14.934, *P* < 0.001, *η*_p_^2^ = 0.104; *P* > 0.084 for all other traits). Visual examination revealed positive relationships between PC1 and both female lean mass and fecundity, while the relationships between PC1 on one side and female fat content, embryo lean mass and RA on the other side were negative (Fig. 6f-i). Similarly, female fat content and fecundity had a positive relationship with PC2 while PC2 and embryo lean mass had a negative relationship (Fig. 7f-i). This means that female lean mass and fecundity increased with increasing water temperature, DO and salinity, but that female fat content, embryo mass and RA decreased along the same axis. Moreover, female fat content and fecundity increased with increasing turbidity, while embryo lean mass decreased. Based on our Mantel tests, neither male life-history distance (*r* = 0.024, *P* = 0.442) nor female life-history distance (*r* = -0.234, *P* = 0.875) were correlated with genetic distance between population pairs.

## Discussion

We studied biogeography as well as population-genetic and life-history differentiation in a widespread fish species, the Sailfin molly (*P. latipinna*), and found very clear genetic structuring that is in general agreement with isolation by distance (IBD). Abiotic factors measured as a snapshot of the conditions we encountered during the collections did not appear to correlate with the distribution of the detected genetic clusters. In addition, life-history trait differentiation showed some congruence with patterns of genetic differentiation, but large proportions of the variation likely respond more strongly to local (i.e., site-specific), rather than cluster-specific, ecological factors.

### Genetic differentiation and biogeography

Below we are evaluating the evidence we provide relative to three important concepts: isolation by distance (IBD), dispersal within paleodrainages and potential human-assisted dispersal. Overall, we have strong support for IBD, indicating reduced genetic connectivity among populations separated by longer distances due to limited dispersal. That seems to be the most “natural” scenario for a species like *P. latipinna*. Although they can tolerate marine conditions, they do not seem to be able to actively swim long distances. However, in some cases as indicated by our migration analyses, sampling localities separated by distinct paleorivers, and considerable distances had or have recent gene flow. Those connections do not align with the expected alternative explanation of dispersal via paleorivers. Altogether, our genetic findings suggest constrained long-distance dispersal in *P. latipinna* and connections between distant populations may not have been facilitated by past river connections but rather by relatively recent dispersal events. The fact that first generation migrants were frequently detected between paleoriver systems suggests that human-assisted dispersal, potentially combined with rare natural events with less predictable outcomes, such as hurricanes, tornadoes, and zoochory, may have shaped the genetic structure of *P. latipinna*.

In trying to dissect potential mechanisms for the pattern we detected, we noted that the genetic composition of populations west of Alabama (including the Mississippi) within the Orange cluster is remarkably homogeneous. The only exception was the population from Brownsville, Texas (LPB). This homogeneous pattern might be a consequence of unhindered migration by individuals via the “Intracoastal Waterway”, a ship channel that connects Brownsville in South Texas with New Orleans and the Mississippi delta. The channel continues to Carrabelle, Florida, but the eastern part is separated by the Harvey Lock in New Orleans, which may be impassible to Sailfin mollies. The Intracoastal Waterway, recognized as migration conduit for various fish species (including two species of *Fundulus* and the Least Killifish, *Heterandria formosa*), has also been suggested to have played a role in the northward expansion of the Amazon molly (*P. formosa*), a congener of the Sailfin molly [72]. Sailfin mollies are likely capable of moving from river to river via the ocean due to their tolerance of high salinity [73], but the ship channel might allow them to move between areas without having to enter marine environments. In the ocean, near-shore currents are expected to drift any Sailfin mollies downcoast [74] in a western and southern direction but these near-shore currents exhibit considerable variability [75]. Movement of livebearing fishes across the ocean has been suggested in scenarios of colonization of islands in the Caribbean (e.g [76]). . , , and, due to sperm storage (allowing a female to have multiple broods without mating again), a single inseminated female could be capable of establishing a new population. This has been demonstrated in a close relative, the guppy (*Poecilia reticulata*) [77].

Why the Brownsville, Texas (LPB) population is different from the other populations west of Alabama (the Orange cluster) is unclear, but the admixture in this population may be influenced by genotypes occurring to the south, in Mexico, which we could not sample for this study. This, too, might be caused by a near-shore current that would transport Sailfin mollies northward up to the mouth of the Nueces River, where this current collides with a southward flowing current from Louisiana [30, 78]. Such dynamic river networks may be a plausible conduit for stepping-stone dispersal.

More generally, while the homogeneous population structure west of Alabama is surprising, albeit potentially explained by the Intracoastal Waterway, the situation east of Alabama is much more complex. Here, as well, patterns are not easily explained by river drainages. In fact, palaeodrainages do not line up with the detected population structuring. The complex situation in Florida thus requires considering several alternative mechanisms of dispersal. Generally, the coastal populations starting in the Florida panhandle (red), continuing down the coast (green) and along the Atlantic coast (light blue) seem to reflect movement of genotypes along the coast in agreement with the predominant marine current systems in the area [79]. On the west coast, currents would mostly transport any Sailfin mollies southward after leaving the river mouths [80, 81]. This is in agreement with the slow fading of Red genotypes as we look at the four populations from West Florida WF1–4). Notably, WF4 and WF5 are in different drainage systems, yet quite similar in genotype composition. On the east coast, currents would take Sailfin mollies northward, potentially explaining the dominance of the light blue genotype along that coast. However, there is also a strong presence of the dark green genotype, which is predominantly associated with the west coast. It is not clear how we can easily account for this, but several mechanisms seem plausible, including currents that flow from the Gulf of Mexico around the southern tip of Florida into the Atlantic [79]. Furthermore, river capture, a geological process, could connect previously separated streams facilitating the movement of aquatic organisms from drainage to drainage during flooding events (e.g., heavy rains, typically caused by hurricanes [43]. The very flat topography of Florida makes such flooding a plausible avenue for fish movement. Several studies have provided evidence that changing water levels in Florida (such as during flooding or drought) are a major driver of fish movement and dispersal, including movement of sailfin mollies and other poeciliid fishes [82, 83]. In fact, Florida is frequently hit by hurricanes [43], both on the Atlantic and the Gulf coasts. Dispersal aided by hurricanes has been suggested specifically for Sailfin mollies based on populations sampled before and after a hurricane [43]. However, it is difficult to attribute dispersal directly to individual events. Our samples were collected in 2008. During the hurricane season the year before, 2007, Tropical Storm ‘Barry’ passed over Florida - a potential influence on the fish migration [84]. Even if hurricanes may not move fishes directly, tornados, which originate over land, are capable of this and may contribute to the dispersal patterns we detected.

Of course, direct natural movement from populations in Florida, cannot be ruled out although it seems unlikely given the distances and the established pattern of isolation by distance. As seen in our migration analysis, transport of fishes by humans is likely to have played a role in the distribution of *P. latipinna* lineages. Sailfin mollies are popular as bait fishes and historically Sailfin mollies have been introduced from Florida or Louisiana by human activity to Central Texas in the San Marcos area, where populations have persisted since the 1950’s [35]. Our finding that Sailfin mollies may have moved along the ship channel reinforces the idea that the current genetic structuring in *P. latipinna* is not organized along river drainages or their past connections, but by a combination of natural and human-facilitated dispersal events. Examples of humans aiding the spread of small fishes as bait or for mosquito control are well established for Sailfin mollies and other species, including guppies (*Poecilia reticulata*) [77, 85] and mosquitofish (genus *Gambusia*) [86–88] and we suggest that this is more widespread than currently appreciated.

The situation in Central Florida is also quite interesting: two inland populations (CF1 and CF4) are very homogenous and represent their own cluster (dark-Blue). Notably, though, they are from two different river drainages. The southern of the two populations is in the same drainage as EF1 from the east coast, but there is no significant sharing of genotypes. The two populations south of them (CF2 and CF3) are mainly composed of the light-Blue genotype, linking them to populations from the East Coast. Together this seems to indicate limited movements in inland populations of *P. latipinna*, while the coastal populations are more connected.

Overall, the notable mismatch between palaeodranainages and the main genetic clusters in *P. latipinna* is in stark contrast to some recent findings on coastal fish biogeography, where paleorivers largely explained the distribution of the main patterns of genetic structure [64, 89, 90]. However, these previous studies were conducted on strictly freshwater species. Sailfin mollies differ from those species in several regards, which could help explain the different patterns we found in our study. First, given its euryhaline nature [31], *P. latipinna* may be and have been able to disperse between river mouth areas, connecting populations that are now shared among currently isolated rivers. Alternatively, human-induced fish movement may have also contributed to the sharing of genetic clusters among paleoriver systems. After all, *P. latipinna* is widely used in the aquarium trade, as bait fish, and for mosquito control [37]. Finally, river capture and/or flooding events may have connected some inland populations (such as CF1 and CF4 in Central Florida).

### Life-history differentiation and geography

While there was considerable genetic structuring, only weak structuring was detected in our life-history analyses, and we did not find any correlation between pairwise-population genetic distances (via *F*_ST_) and pairwise-population life-history distance in either sex (via Euclidean distances). Nonetheless, there were many differences in specific traits between genetic clusters. Most of these differences were idiosyncratic (i.e., not following clear patterns suggesting overarching clines between clusters) so that for different traits (both per sex but also across both sexes) it was usually different sets of genetic clusters that differed most from one another. Further, complex life-history traits are likely polygenic and subject to environmental influence (phenotypic plasticity). Nonetheless, these life-history differences between genetic clusters could indicate that at least some of the neutral genetic differentiation we uncovered might be mirrored by functional genetic differentiation reflecting local adaptation.

The only tangible signal we uncovered was for embryo lean mass, for which values were smallest for populations originating from the Western Gulf of Mexico Region (Cluster 1), intermediate for West and East Coast populations of Florida (Clusters 2, 3, 4), and greatest for populations from Central Florida (Clusters 4 and 5). Offspring size is known to respond to resource availability, competition, and predation, with offspring size usually being lower in high-resource, low-competition or high-predation environments [91, 92], but these selective pressures can interact in complex ways (e.g [92, 93]). This suggests the presence of large geographic gradients in predation, population density, and/or resource availability between each of these clusters and localities. For example, together with the fact that male size was also largest in sites from Central Florida, this could suggest that the inland populations in Florida might experience less predation than the coastal populations of Florida and the western Gulf region. Nonetheless, we currently lack the data to fully interpret this and call on future studies to investigate this further.

However, if we explore life-history patterns by themselves and in relation to local environmental variables, we detect several interesting patterns. First, in natural populations, male size of many Poeciliids is bimodally distributed with many small and large males but relatively few intermediate-sized ones [69, 70, 94], and it has been shown for swordtails (genus *Xiphophorus*) that this bimodality is genetically controlled via the *P*-locus [69]. For sailfin mollies, previous results had been mixed with evidence of bimodality from some populations but not from others [17]. In our data, we did not find a single population with male size following a bimodal distribution, a pattern mirrored in some other mollies (e.g., *Poecilia mexicana*: [95], and *Limia perugiae* [96]). This might suggest that male size is not under the kind of genetic control described for other Poeciliids but could also simply be the result of environmental effects overriding potential genetic determination [94] or of populations being fixed for one allele at the *P*-locus.

It was no surprise that male and female lean mass, but also female fecundity, increased with fish SL (longer fish are heavier; larger females also ought to have more body cavity space to accommodate more developing offspring). However, GSI decreased with increasing male SL, likely due to the sailfin molly mating system. Smaller males usually invest less into male secondary characteristics (i.e., bold body and fin coloration and elaborate dorsal fins) and follow a sneaker tactic that relies on approaching females from behind and attempting to force copulations via gonopodial thrusting [97–99]. Large males, on the other hand, are usually very colorful, have elaborate dorsal fins, and rely on male courtship displays to attract female attention [98, 100], with females generally preferring large over small males [46, 99]. Smaller males investing more into relative testis size than larger males to facilitate their mating behavior of forced copulations makes intuitively sense and has been found before [101].

We found RA and embryo fat content to decrease with increasing developmental stage. Both of these patterns have been well documented in poeciliid fishes [102, 103]. Sailfin mollies are predominantly lecithotrophic species (i.e., almost all resources needed for embryo development are usually stored in the yolk prior to fertilization), although they can supplement embryos with additional nutrients under some environmental conditions [102, 104]. This means that embryos will lose roughly 40% of their mass during embryo development, which will result in a lower RA towards the end of pregnancy compared to RA closer to fertilization [102]. Similarly, embryonic fat content usually also declines with increasing embryonic stage [67, 103], probably because fat reserves in the yolk get metabolized by the embryo during development.

With respect to environmental variables, male and female lean mass as well as fecundity increased with increasing water temperature, oxygen content, and salinity, while male and female fat content, embryo lean mass, and RA decreased along the same gradients. Male lean mass and fat content also increased with increasing turbidity and pH, while female fat content and fecundity increased with increasing turbidity, but embryo lean mass decreased along the same gradient. Greater lean mass as a function of increasing salinity has been found in sailfin mollies and other Poeciliids before (sailfin mollies: [17]; *Gambusia* spp.: [91]; *Phalloptychus januarius*: [28] and could be a reflection of increased ionoregulatory advantages of a larger body mass [105, 106] and other physiological effects of salinity. Similarly, the effects of temperature on fish size (SL and/or mass) have been reported for other poeciliid fishes before (*Gambusia affinis*: [107]; *Gambusia puncticulata*: [108], and an experimental positive link between increased temperature and fish growth has also been documented [107] even though, on a global scale, responses to temperature in fish can go in either direction [109].

The effects of DO and turbidity on some of the traits are more difficult to explain. Nonetheless, given that DO co-varied with temperature and salinity, the effect of DO could be due to that covariation rather than an indication of direct responses to DO. Increasing turbidity in aquatic habitats is usually accompanied by reduced primary productivity [110, 111], so we would therefore expect fish in more turbid waters to be smaller and have less fat reserves; yet we found the opposite pattern. Behaviorally, turbidity increases the time needed for mate choice in Sailfin mollies [112]. However, turbidity may correlate with other environmental variables we did not capture, and the responses we observed could be influenced by these other variables, rather than turbidity itself. For example, it is possible that some measured turbidity was due to high algal biomass rather than suspended inorganic particles [113]. If so, greater turbidity would correlate with habitat productivity, resulting in the observed patterns. Overall, environmental patterns may be influenced by seasonality, however, to control for this we conducted the collections during a few weeks in the summer, when conditions are relatively stable.

It appears that migration and exchange of genotypes made possible by dispersal from river mouth to river mouth (as suggested by our mismatches between genetic clusters and palaeoriver systems) and possible human-mediated dispersal provide the glue that holds this species together. This interpretation is also in agreement with the relatively large number of recent migrants between sites in different river (and paleoriver) systems.

Overall, we found that genetic differentiation in Sailfin mollies revealed distinct genetic clusters and a general isolation-by-distance population structure, with some expected admixture supported by the several mismatches between paleodrainages and geographical distribution of the genetic clusters. Neither life-history patterns nor the abiotic factors we recorded provided strong explanations for the observed genetic structuring. Instead, despite the large distribution range and apparently limited dispersal capabilities, there might be sufficient gene flow to hinder cryptic speciation in this widespread species.

### Animal ethics declaration

This project was approved by University of Oklahoma IACUC #R05-001. Collecting permits were granted by various state agencies. Texas Scientific Permit Number SPR-0305-045; Louisiana Scientific Collecting Permit S-81-2008; South Carolina Scientific Collecting Permit F-08-30; Georgia Scientific Collecting Permit 29-WBH-08-148; Florida License 580-957-322; Alabama Permit 4607. Collections in Mississippi were conducted under a permit issued to Jake Schaefer (University of Southern Mississippi) and IS and the collection in North Carolina was done in collaboration with the NC DNR office in Wilmington.

### Electronic supplementary material

Below is the link to the electronic supplementary material.

Supplementary Material 1

Supplementary Material 2

## Data Availability

The datasets used and/or analysed during the current study are available from the corresponding author on reasonable request. The paleodrainage analysis is avalailable here: https://github.com/waldirmbf/Paleodrainages. Raw data is available on Dryad (DOI: 10.5061/dryad.2jm63xsxb) and as supplementary data attached to this manuscript.
