# Supplementary material for: Genetic and phenotypic diversification in a widespread fish, the Sailfin Molly (Poecilia latipinna)
Source: BMC Ecol Evol. 2024 Jul 1;24:87. doi: 10.1186/s12862-024-02270-x (PMC11218414; doi:10.1186/s12862-024-02270-x)
Supplement: Supplementary file 2 — Supplementary Material 2 [file 12862_2024_2270_MOESM2_ESM.docx]

Supplementary material

**Genetic and phenotypic diversification in a widespread fish, the Sailfin molly (*Poecilia latipinna*)**

Ralph Tiedemann, Rüdiger Riesch, Maxi Tomowski, Katja Havenstein, Jan Schlupp, Waldir M. Berbel-Filho & Ingo Schlupp

**Supplementary Background Information**

Sailfin mollies are members of the family Poeciliidae, are livebearing, show ovoviviparity, and have internal fertilization [1]. As is typical for the family, males possess a modified anal fin, the gonopodium. This is used to transfer sperm into the female gonoduct during a short copulation and males make no paternal contribution beyond the ejaculate. The gestation period is ca. 21−30 days and offspring are immediately independent from the mother. In addition, paternity within clutches is often mixed [2-4]. Sailfin molly males are highly ornamented and use two tactics to obtain matings. They may court a female or try to obtain sneak copulations [5, 6]. Males can switch between these tactics, although the largest males of any given population mostly court, while the smallest males almost always attempt forced copulations [7]. In livebearing fishes, such as the Sailfin molly, females are on average larger than males, reaching maturity around 30mm and growing indeterminately to more than 80mm [8]. Males effectively cease growth at maturity [9]. They can reach maturity as small as 18mm, but also reach a size of up to 80mm [10]. Small males are typically much more common than larger males [11].

Phylogenetically, the Sailfin molly forms a clade with other mollies showing a long dorsal fin. This clade is part of the genus *Poecilia* (formerly *Mollienesia*), which in turn is part of the family Poeciliidae [12-14].

Furthermore, between the Panuco basin in Tamaulipas, Mexico and the Nueces River, Texas, USA, Sailfin mollies are the sexual hosts to a unisexual, sperm-parasitic fish of hybrid origin, the Amazon molly, *Poecilia formosa* [15].

**Supplementary Results** (of preliminary life-history analyses)

The GLM on male SL revealed significant effects of both independent variables [Genetic Cluster: *F*_5,181_ = 6.973, *P* < 0.001; Population(Genetic Cluster): *F*_8,181_ = 5.405, *P* < 0.001], but based on our measure of effect size (partial eta squared, *η*_p_^2^) variation between populations within genetic clusters was slightly more important (*η*_p_^2^ = 0.193) than variation due to genetic cluster (0.163). We uncovered a parallel pattern in the GLM of female SL [Genetic Cluster: *F*_4,128_ = 2.745, *P* = 0.031; Population(Genetic Cluster): *F*_5,128_ = 19.322, *P* < 0.001], albeit with a much stronger effect size for variation amongst populations within genetic clusters (*η*_p_^2^ = 0.430) than for variation amongst genetic clusters (*η*_p_^2^ = 0.079).

The multivariate GLM on the remaining male traits returned significant effects of the covariate SL (*F*_3,178_ = 3873.374, *P* < 0.001), but also of Genetic Cluster (*F*_15,491_ = 6.621, *P* < 0.001) and Population(Genetic Cluster) (*F*_24,517_ = 12.443, *P* < 0.001). Nonetheless, based on *η*_p_^2^, SL had by far the strongest effect (0.985), while the effect of Population(Genetic Cluster) was much weaker (0.357) but still more important than that of Genetic Cluster (0.156). Post-hoc univariate comparisons (Bonferroni-corrected significance at ⍺ = 0.017) demonstrated that SL significantly affected male lean mass (*F*_1,180_ = 11491.200, *P* < 0.001, *η*_p_^2^ = 0.985) and GSI (*F*_1,180_ = 45.709, *P* < 0.001, *η*_p_^2^ = 0.203), but not fat content (*F*_1,180_ = 2.201, *P* = 0.140, *η*_p_^2^ = 0.012). While male lean mass increased with increasing SL, GSI decreased with increasing SL so that larger males had smaller relative testis mass. Genetic Cluster and Population(Genetic Cluster), on the other hand, significantly affected all three traits (Genetic Cluster, lean mass: *F*_5,180_ = 5.350, *P* < 0.001, *η*_p_^2^ = 0.129; fat content: *F*_5,180_ = 8.213, *P* < 0.001, *η*_p_^2^ = 0.186; GSI: *F*_5,180_ = 5.169, *P* < 0.001, *η*_p_^2^ = 0.126; Population(Genetic Cluster), lean mass: *F*_8,180_ = 25.807, *P* = <0.001, *η*_p_^2^ = 0.534; fat content: *F*_8,180_ = 9.932, *P* < 0.001, *η*_p_^2^ = 0.306; GSI: *F*_8,180_ = 4.075, *P* < 0.001, *η*_p_^2^ = 0.153).

The multivariate GLM on the remaining female traits demonstrated significant effects of the covariates SL (*F*_6,121_ = 310.471, *P* < 0.001) and Embryonic Stage of Development (*F*_6,121_ = 8.679, *P* < 0.001), but also of Genetic Cluster (*F*_24,423_ = 7.566, *P* < 0.001) and Population(Genetic Cluster) (*F*_30,486_ = 18.258, *P* < 0.001). Again, SL had by far the strongest effect (*η*_p_^2^ = 0.939), while all other effects were much weaker [Embryonic Stage of Development: 0.301; Genetic Cluster: 0.268; Population(Genetic Cluster: 0.453)], even though the effect of variation between populations within a genetic cluster was again stronger than that of variation between Genetic Clusters. Post-hoc univariate comparisons (Bonferroni-corrected significance at ⍺ = 0.008) demonstrated that SL significantly affected female lean mass (*F*_1,126_ = 1757.468, *P* < 0.001, *η*_p_^2^ = 0.933), fecundity (*F*_1,126_ = 103.535, *P* < 0.001, *η*_p_^2^ = 0.451) and embryo lean mass (*F*_1,126_ = 7.606, *P* = 0.007, *η*_p_^2^ = 0.057), while the other traits were not significantly affected (*P* > 0.317 in all cases). SL had a positive relationship with all three variables, so that larger females had greater lean mass and fecundity and produced larger offspring. Embryonic Stage of Development had significant effects on female fat content (*F*_1,126_ = 9.533, *P* = 0.002, *η*_p_^2^ = 0.070), embryo fat content (*F*_1,126_ = 35.843, *P* < 0.001, *η*_p_^2^ = 0.221), embryo lean mass (*F*_1,126_ = 7.952, *P* = 0.006, *η*_p_^2^ = 0.059) and RA (*F*_1,126_ = 12.313, *P* < 0.001, *η*_p_^2^ = 0.089), but not on the other two traits (*P* > 0.386 in both cases). Genetic Cluster, on the other hand, significantly affected all traits (lean mass: *F*_4,126_ = 4.902, *P* = 0.001, *η*_p_^2^ = 0.135; fat content: *F*_4,126_ = 12.458, *P* < 0.001, *η*_p_^2^ = 0.283; fecundity: *F*_4,126_ = 13.398, *P* < 0.001, *η*_p_^2^ = 0.298; embryo fat content: *F*_4,126_ = 4.355, *P* = 0.002, *η*_p_^2^ = 0.121; embryo lean mass: *F*_4,126_ = 14.486, *P* < 0.001, *η*_p_^2^ = 0.315; RA: *F*_4,126_ = 4.193, *P* = 0.003, *η*_p_^2^ = 0.117) and the same was true for Population(Genetic Cluster) (lean mass: *F*_5,126_ = 32.441, *P* < 0.001, *η*_p_^2^ = 0.563; fat content: *F*_5,126_ = 33.091, *P* < 0.001, *η*_p_^2^ = 0.568; fecundity: *F*_5,126_ = 14.435, *P* < 0.001, *η*_p_^2^ = 0.364; embryo fat content: *F*_5,126_ = 5.817, *P* < 0.001, *η*_p_^2^ = 0.188; embryo lean mass: *F*_5,126_ = 29.450, *P* < 0.001, *η*_p_^2^ = 0.539; RA: *F*_5,126_ = 9.686, *P* < 0.001, *η*_p_^2^ = 0.278).

**Supplementary Discussion** (of analyses presented in the main manuscript)

It was no surprise that male and female lean mass, but also female fecundity, increased with fish SL (longer fish are heavier; larger females also ought to have more body cavity space to accommodate more developing offspring). However, GSI decreased with increasing male SL, likely due to the sailfin molly mating system. Smaller males usually invest less into male secondary characteristics (i.e., bold body and fin coloration and elaborate dorsal fins) and follow a sneaker tactic that relies on approaching females from behind and attempting to force copulations via gonopodial thrusting [7, 16, 17]. Large males, on the other hand, are usually very colorful, have elaborate dorsal fins, and rely on male courtship displays to attract female attention [16, 18], with females generally preferring large over small males [17, 19]. Smaller males investing more into relative testis size than larger males to facilitate their mating behavior of forced copulations makes intuitively sense. We found RA and embryo fat content to decrease with increasing developmental stage. Both of these patterns have been well documented in poeciliid fishes [20, 21]. Sailfin mollies are predominantly lecithotrophic species (i.e., almost all resources needed for embryo development are usually stored in the yolk prior to fertilization), although they can supplement embryos with additional nutrients under some environmental conditions [20, 22]. This means that embryos will lose roughly 40% of their mass during embryo development, which will result in a lower RA towards the end of pregnancy compared to RA closer to fertilization [20]. Similarly, embryonic fat content usually also declines with increasing embryonic stage [23] [21], probably because fat reserves in the yolk get metabolized by the embryo during development.

Supplementary Table 1: Specifications for the Hydrolab probe (Datasonde 4a) used.

| **Parameter** | **Range** | **Accuracy** | **Resolution** |
| --- | --- | --- | --- |
| Temperature | -5−50 Celsius | ±.10 | 0.01 |
| Specific Conductivity | 0−100 mS/cm | ± 1% of reading  ± 0.001 mS/cm | 4 digits |
| pH | 0−14 units | ± 0.2 units | 0.01 units |
| Dissolved Oxygen (DO) | 0−50 mg/L | ± 0.2 mg/L | 0.01 mg/L |
| Turbidity (Shuttered) | 0−100 or 100−1000 NTU | ± 2.6% of range 0.1 or 1 NTU |  |
| Chlorophyll a | 0.02 microgr/L | 0.01 microgr/L |  |

Supplementary Table 2: Sampling locations

| **Field Number** | **ID** | **Geographic Location** |
| --- | --- | --- |
| IS 08063 | LPB | Lincoln Park, Brownsville (South-Texas); N 25° 53.976 W 097° 28.763 |
| IS 08011 | NET | TX 124787; N 29° 33.101 W 094° 23.343 |
| IS 08012 | CAM | Cameron, LA, HWY 82 (Louisiana); N 29° 45.631 W 093° 51.016 |
| IS 08015 | LA1 | HWY 14 + HWY 331; N 29° 57.517 W 092° 01.278 |
| IS 08021 | LA2 | HW 90, LA; N 30° 14.281 W 89° 37.228 |
| IS 08024 | MIS | HWY 613 (Mississippi); N 30° 30.720 W 088° 33.073 |
| IS 08030 | WF1 | HWY 98, Florida near Royal Bluff; N 29° 47.872 W 84° 44.678 |
| IS 08031 | WF2 | Panacea Mineral Spring Pool, Panacea, FL; N 30° 02.069 W 84° 23.389 |
| IS 08047 | CF1 | Florida Inland; N 29° 38.069 W 82° 20.330 |
| IS 08038 | WF4 | Roadside Ditch, HWY 595 (Florida W); N 28° 29.925 W 082° 38.865 |
| IS 08039 | WF5 | Graveyard Ditch, (Florida W); N 27° 50.945 W 082° 40.076 |
| IS 08041 | CF2 | Roadside Ditch Haines City, FL (Florida Inland); N 28° 06.845 W 081° 36.239 |
| IS 08042 | CF3 | Roadside Ditch Kissimmee, FL; N 28° 08.745 W 081° 27.968 |
| IS 08043 | EF1 | Roadside Ditch Satellite Beach (Florida East); N 28° 10.341 W 80° 35.780 |
| IS 08046 | CF4 | Boatramp near Lake Enstins, FL (Florida Inland); N 28° 51.045 W 081° 41.423 |
| IS 08048 | EF2 | Boatramp Bulow Creek, Volusia County, FL (Florida East); N 29° 24.437 W 81° 07.322 |
| IS 08049 | EF3 | Six Mile Landing (Florida East); N 30° 06.00 W 081° 20.695 |
| IS 08055 | SC1 | James Island County Park near Charleston, SC; N 32° 44.047 W 79° 59.755 |

Supplementary Table 3: Microsatellite data per locus and population. P <0.05 indicates deviation of allele frequencies from Hardy-Weinberg-equilibrium (i.e., HO = HE).

| **Locus** | **Population** |  | **N** | **No. alleles** | **Allele size** | **H_0_** | **H_E_** | **P** | **A_R_** |
| --- | --- | --- | --- | --- | --- | --- | --- | --- | --- |
| **GT-I41** | PlatIS08062 | LPB | 10 | 3 | 155-159 | 0.400 | 0.358 | 1.000 | 2.526 |
|  | PlatIS08011 | NET | 10 | 2 | 155-157 | 0.200 | 0.189 | 1.000 | 1.763 |
|  | PlatIS08012 | CAM | 10 | 2 | 155-157 | 0.600 | 0.505 | 1.000 | 2.000 |
|  | PlatIS08015 | LA1 | 10 | 2 | 155-157 | 0.700 | 0.479 | 0.220 | 1.998 |
|  | PlatIS08021 | LA2 | 10 | 2 | 155-157 | 0.500 | 0.395 | 1.000 | 1.984 |
|  | PlatIS08024 | MIS | 10 | 2 | 155-157 | 0.100 | 0.100 | 1.000 | 1.500 |
|  | PlatIS08030 | WF1 | 10 | 3 | 157-167 | 0.700 | 0.511 | 0.631 | 2.495 |
|  | PlatIS08031 | WF2 | 8 | 3 | 157-163 | 0.750 | 0.675 | 0.158 | 2.964 |
|  | PlatIS08038 | WF4 | 10 | 3 | 157-163 | 0.300 | 0.279 | 1.000 | 2.263 |
|  | PlatIS08039 | WF5 | 10 | 4 | 151-167 | 0.700 | 0.605 | 1.000 | 3.351 |
|  | PlatIS08043 | EF1 | 10 | 3 | 157-161 | 0.500 | 0.584 | 0.102 | 2.878 |
|  | PlatIS08048 | EF2 | 9 | 4 | 153-161 | 0.444 | 0.399 | 1.000 | 2.928 |
|  | PlatIS08049 | EF3 | 10 | 1 | 157 | - | - | - | 1.000 |
|  | PlatIS08041 | CF2 | 6 | 2 | 157-159 | 0.167 | 0.167 | 1.000 | 1.833 |
|  | PlatIS08042 | CF3 | 5 | 2 | 157-159 | 0.200 | 0.467 | 0.333 | 2.000 |
|  | PlatIS08046 | CF4 | 10 | 2 | 157-159 | 0.300 | 0.395 | 0.480 | 1.984 |
|  | PlatIS08047 | CF1 | 10 | 1 | 157 | - | - | - | 1.000 |
|  | PlatIS08055 | SC1 | 10 | 1 | 157 | - | - | - | 1.000 |
| **GA-IV42** | PlatIS08062 | LPB | 10 | 6 | 174-202 | 0.900 | 0.853 | 0.996 | 5.187 |
|  | PlatIS08011 | NET | 10 | 2 | 174-198 | 0.300 | 0.268 | 1.000 | 1.895 |
|  | PlatIS08012 | CAM | 10 | 2 | 174-198 | 0.400 | 0.337 | 1.000 | 1.957 |
|  | PlatIS08015 | LA1 | 10 | 3 | 174-198 | 0.400 | 0.468 | 1.000 | 2.484 |
|  | PlatIS08021 | LA2 | 10 | 2 | 174-198 | 0.500 | 0.479 | 1.000 | 1.998 |
|  | PlatIS08024 | MIS | 10 | 2 | 174-198 | 0.200 | 0.337 | 0.306 | 1.957 |
|  | PlatIS08030 | WF1 | 10 | 2 | 196-198 | 0.200 | 0.189 | 1.000 | 1.763 |
|  | PlatIS08031 | WF2 | 8 | 7 | 176-200 | 1.000 | 0.842 | 0.840 | 5.455 |
|  | PlatIS08038 | WF4 | 10 | 8 | 182-202 | 0.800 | 0.884 | 0.122 | 6.028 |
|  | PlatIS08039 | WF5 | 10 | 8 | 174-202 | 0.900 | 0.863 | 0.502 | 5.872 |
|  | PlatIS08043 | EF1 | 10 | 11 | 180-266 | 1.000 | 0.916 | 1.000 | 7.168 |
|  | PlatIS08048 | EF2 | 9 | 12 | 186-260 | 0.889 | 0.941 | 0.562 | 7.873 |
|  | PlatIS08049 | EF3 | 10 | 9 | 180-242 | 0.900 | 0.826 | 0.913 | 5.921 |
|  | PlatIS08041 | CF2 | 6 | 5 | 186-242 | 0.500 | 0.727 | 0.252 | 4.500 |
|  | PlatIS08042 | CF3 | 5 | 6 | 186-242 | 0.600 | 0.889 | 0.147 | 6.000 |
|  | PlatIS08046 | CF4 | 10 | 8 | 182-226 | 1.000 | 0.884 | 0.958 | 6.063 |
|  | PlatIS08047 | CF1 | 10 | 8 | 190-228 | 1.000 | 0.795 | 0.464 | 5.247 |
|  | PlatIS08055 | SC1 | 10 | 2 | 220-242 | 0.100 | 0.100 | 1.000 | 1.500 |
| **GA-I29B** | PlatIS08062 | LPB | 10 | 8 | 215-243 | 0.800 | 0.805 | 0.594 | 5.351 |
|  | PlatIS08011 | NET | 10 | 4 | 225-231 | 0.600 | 0.574 | 1.000 | 2.995 |
|  | PlatIS08012 | CAM | 10 | 4 | 225-231 | 0.200 | 0.284 | 0.159 | 2.500 |
|  | PlatIS08015 | LA1 | 10 | 3 | 227-231 | 0.700 | 0.542 | 0.280 | 2.498 |
|  | PlatIS08021 | LA2 | 10 | 2 | 227-229 | 0.700 | 0.521 | 0.520 | 2.000 |
|  | PlatIS08024 | MIS | 10 | 3 | 225-229 | 0.500 | 0.637 | 0.635 | 2.893 |
|  | PlatIS08030 | WF1 | 10 | 6 | 225-247 | 0.500 | 0.826 | 0.032 | 4.961 |
|  | PlatIS08031 | WF2 | 8 | 6 | 221-255 | 0.625 | 0.767 | 0.193 | 4.742 |
|  | PlatIS08038 | WF4 | 10 | 9 | 227-257 | 0.900 | 0.868 | 0.822 | 6.109 |
|  | PlatIS08039 | WF5 | 10 | 11 | 219-257 | 0.900 | 0.932 | 0.285 | 7.404 |
|  | PlatIS08043 | EF1 | 10 | 5 | 229-263 | 0.700 | 0.621 | 0.771 | 3.789 |
|  | PlatIS08048 | EF2 | 9 | 12 | 221-263 | 0.889 | 0.941 | 0.574 | 7.873 |
|  | PlatIS08049 | EF3 | 10 | 11 | 227-265 | 1.000 | 0.937 | 0.667 | 7.474 |
|  | PlatIS08041 | CF2 | 6 | 3 | 227-247 | 0.833 | 0.727 | 0.668 | 3.000 |
|  | PlatIS08042 | CF3 | 5 | 4 | 229-255 | 0.400 | 0.733 | 0.114 | 4.000 |
|  | PlatIS08046 | CF4 | 10 | 4 | 225-243 | 0.300 | 0.489 | 0.168 | 2.957 |
|  | PlatIS08047 | CF1 | 10 | 7 | 225-253 | 0.600 | 0.805 | 0.031 | 4.939 |
|  | PlatIS08055 | SC1 | 10 | 9 | 231-267 | 0.900 | 0.900 | 0.915 | 6.466 |
| **GA-III29B** | PlatIS08062 | LPB | 10 | 11 | 214-254 | 0.900 | 0.916 | 0.828 | 7.168 |
|  | PlatIS08011 | NET | 10 | 6 | 236-264 | 0.800 | 0.811 | 0.718 | 4.807 |
|  | PlatIS08012 | CAM | 10 | 9 | 230-252 | 0.800 | 0.826 | 0.689 | 5.921 |
|  | PlatIS08015 | LA1 | 10 | 7 | 236-250 | 0.700 | 0.774 | 0.334 | 5.053 |
|  | PlatIS08021 | LA2 | 10 | 7 | 216-248 | 1.000 | 0.879 | 0.970 | 5.791 |
|  | PlatIS08024 | MIS | 10 | 8 | 228-244 | 0.900 | 0.868 | 0.986 | 5.942 |
|  | PlatIS08030 | WF1 | 10 | 5 | 202-228 | 0.500 | 0.505 | 0.649 | 3.395 |
|  | PlatIS08031 | WF2 | 8 | 9 | 212-240 | 0.875 | 0.917 | 0.700 | 6.992 |
|  | PlatIS08038 | WF4 | 10 | 7 | 212-242 | 1.000 | 0.858 | 0.997 | 5.635 |
|  | PlatIS08039 | WF5 | 10 | 10 | 214-236 | 0.900 | 0.889 | 0.568 | 6.679 |
|  | PlatIS08043 | EF1 | 10 | 7 | 212-256 | 0.800 | 0.832 | 0.852 | 5.198 |
|  | PlatIS08048 | EF2 | 9 | 8 | 214-236 | 0.778 | 0.889 | 0.588 | 6.186 |
|  | PlatIS08049 | EF3 | 10 | 8 | 216-248 | 0.700 | 0.837 | 0.710 | 5.613 |
|  | PlatIS08041 | CF2 | 6 | 6 | 214-240 | 1.000 | 0.879 | 0.764 | 5.636 |
|  | PlatIS08042 | CF3 | 5 | 5 | 214-230 | 0.800 | 0.822 | 0.901 | 5.000 |
|  | PlatIS08046 | CF4 | 10 | 8 | 218-242 | 0.600 | 0.742 | 0.031 | 5.158 |
|  | PlatIS08047 | CF1 | 10 | 6 | 214-236 | 0.700 | 0.721 | 0.305 | 4.483 |
|  | PlatIS08055 | SC1 | 10 | 7 | 214-238 | 1.000 | 0.863 | 0.893 | 5.555 |
| **GA-V18** | PlatIS08062 | LPB | 10 | 4 | 115-145 | 0.600 | 0.679 | 0.723 | 3.440 |
|  | PlatIS08011 | NET | 10 | 4 | 123-129 | 0.200 | 0.363 | 0.106 | 2.763 |
|  | PlatIS08012 | CAM | 10 | 5 | 117-129 | 0.400 | 0.442 | 0.220 | 3.263 |
|  | PlatIS08015 | LA1 | 10 | 4 | 121-133 | 0.600 | 0.500 | 1.000 | 3.158 |
|  | PlatIS08021 | LA2 | 10 | 5 | 121-129 | 0.900 | 0.774 | 0.947 | 4.404 |
|  | PlatIS08024 | MIS | 10 | 5 | 119-129 | 0.900 | 0.800 | 1.000 | 4.535 |
|  | PlatIS08030 | WF1 | 10 | 4 | 115-129 | 0.600 | 0.668 | 0.576 | 3.389 |
|  | PlatIS08031 | WF2 | 8 | 4 | 115-131 | 0.500 | 0.442 | 1.000 | 3.125 |
|  | PlatIS08038 | WF4 | 10 | 6 | 115-137 | 0.700 | 0.705 | 1.000 | 3.999 |
|  | PlatIS08039 | WF5 | 10 | 7 | 115-133 | 0.700 | 0.811 | 0.332 | 4.946 |
|  | PlatIS08043 | EF1 | 10 | 5 | 109-129 | 0.800 | 0.800 | 0.810 | 4.535 |
|  | PlatIS08048 | EF2 | 9 | 7 | 109-143 | 0.889 | 0.843 | 0.296 | 5.402 |
|  | PlatIS08049 | EF3 | 10 | 7 | 115-137 | 0.600 | 0.689 | 0.135 | 4.658 |
|  | PlatIS08041 | CF2 | 6 | 1 | 115 | - | - | - | 1.000 |
|  | PlatIS08042 | CF3 | 5 | 4 | 109-139 | 0.400 | 0.644 | 0.237 | 4.000 |
|  | PlatIS08046 | CF4 | 10 | 3 | 109-121 | 0.700 | 0.568 | 0.541 | 2.758 |
|  | PlatIS08047 | CF1 | 10 | 4 | 121-167 | 0.300 | 0.284 | 1.000 | 2.500 |
|  | PlatIS08055 | SC1 | 10 | 4 | 115-123 | 0.600 | 0.647 | 0.252 | 3.413 |
| **GA-III49A** | PlatIS08062 | LPB | 10 | 5 | 404-418 | 0.600 | 0.568 | 0.275 | 3.658 |
|  | PlatIS08011 | NET | 10 | 7 | 404-426 | 0.900 | 0.858 | 0.575 | 5.635 |
|  | PlatIS08012 | CAM | 10 | 8 | 400-422 | 0.800 | 0.889 | 0.676 | 6.194 |
|  | PlatIS08015 | LA1 | 10 | 10 | 394-422 | 0.900 | 0.868 | 0.964 | 6.551 |
|  | PlatIS08021 | LA2 | 10 | 7 | 402-420 | 0.900 | 0.811 | 0.868 | 5.315 |
|  | PlatIS08024 | MIS | 10 | 4 | 402-418 | 0.300 | 0.284 | 1.000 | 2.500 |
|  | PlatIS08030 | WF1 | 10 | 5 | 402-426 | 0.400 | 0.368 | 1.000 | 3.000 |
|  | PlatIS08031 | WF2 | 8 | 7 | 398-422 | 0.875 | 0.900 | 0.421 | 6.143 |
|  | PlatIS08038 | WF4 | 10 | 10 | 402-434 | 1.000 | 0.884 | 0.679 | 6.494 |
|  | PlatIS08039 | WF5 | 10 | 7 | 402-422 | 0.900 | 0.842 | 1.000 | 5.399 |
|  | PlatIS08043 | EF1 | 10 | 7 | 404-416 | 0.900 | 0.874 | 0.479 | 5.756 |
|  | PlatIS08048 | EF2 | 9 | 8 | 400-436 | 1.000 | 0.850 | 0.992 | 5.832 |
|  | PlatIS08049 | EF3 | 10 | 11 | 402-430 | 1.000 | 0.916 | 0.842 | 7.071 |
|  | PlatIS08041 | CF2 | 6 | 7 | 402-436 | 1.000 | 0.909 | 0.598 | 6.455 |
|  | PlatIS08042 | CF3 | 5 | 7 | 404-436 | 1.000 | 0.933 | 1.000 | 7.000 |
|  | PlatIS08046 | CF4 | 10 | 5 | 410-424 | 0.600 | 0.511 | 1.000 | 3.526 |
|  | PlatIS08047 | CF1 | 10 | 5 | 404-420 | 0.400 | 0.679 | 0.099 | 3.758 |
|  | PlatIS08055 | SC1 | 10 | 5 | 410-426 | 0.800 | 0.737 | 0.704 | 4.142 |
| **GA-II41** | PlatIS08062 | LPB | 10 | 4 | 118-126 | 0.500 | 0.553 | 0.100 | 3.220 |
|  | PlatIS08011 | NET | 10 | 1 | 118 | - | - | - | 1.000 |
|  | PlatIS08012 | CAM | 10 | 1 | 118 | - | - | - | 1.000 |
|  | PlatIS08015 | LA1 | 10 | 1 | 118 | - | - | - | 1.000 |
|  | PlatIS08021 | LA2 | 10 | 2 | 118-120 | 0.100 | 0.100 | 1.000 | 1.500 |
|  | PlatIS08024 | MIS | 10 | 1 | 118 | - | - | - | 1.000 |
|  | PlatIS08030 | WF1 | 10 | 2 | 118-122 | 0.500 | 0.521 | 1.000 | 2.000 |
|  | PlatIS08031 | WF2 | 8 | 2 | 118-122 | 0.250 | 0.400 | 0.385 | 1.992 |
|  | PlatIS08038 | WF4 | 10 | 4 | 118-124 | 0.400 | 0.437 | 0.301 | 3.026 |
|  | PlatIS08039 | WF5 | 10 | 5 | 118-126 | 0.700 | 0.621 | 0.767 | 3.789 |
|  | PlatIS08043 | EF1 | 10 | 1 | 122 | - | - | - | 1.000 |
|  | PlatIS08048 | EF2 | 9 | 2 | 122-124 | 0.111 | 0.111 | 1.000 | 1.556 |
|  | PlatIS08049 | EF3 | 10 | 2 | 122-130 | 0.100 | 0.100 | 1.000 | 1.500 |
|  | PlatIS08041 | CF2 | 6 | 1 | 122 | - | - | - | 1.000 |
|  | PlatIS08042 | CF3 | 5 | 2 | 122-130 | 0.200 | 0.200 | 1.000 | 2.000 |
|  | PlatIS08046 | CF4 | 10 | 2 | 122-124 | 0.200 | 0.189 | 1.000 | 1.763 |
|  | PlatIS08047 | CF1 | 10 | 2 | 120-122 | 0.200 | 0.189 | 1.000 | 1.763 |
|  | PlatIS08055 | SC1 | 10 | 1 | 122 | - | - | - | 1.000 |
| **GA-I47A** | PlatIS08062 | LPB | 10 | 7 | 129-183 | 0.800 | 0.816 | 0.904 | 5.140 |
|  | PlatIS08011 | NET | 10 | 7 | 131-187 | 0.800 | 0.768 | 0.985 | 4.983 |
|  | PlatIS08012 | CAM | 8 | 5 | 135-173 | 0.625 | 0.608 | 0.631 | 4.000 |
|  | PlatIS08015 | LA1 | 10 | 8 | 133-179 | 0.700 | 0.779 | 0.425 | 5.289 |
|  | PlatIS08021 | LA2 | 9 | 11 | 135-189 | 0.778 | 0.856 | 0.362 | 6.817 |
|  | PlatIS08024 | MIS | 10 | 5 | 131-171 | 0.500 | 0.600 | 0.212 | 3.484 |
|  | PlatIS08030 | WF1 | 10 | 4 | 123-137 | 0.500 | 0.611 | 0.422 | 3.483 |
|  | PlatIS08031 | WF2 | 8 | 6 | 123-139 | 0.875 | 0.783 | 1.000 | 4.749 |
|  | PlatIS08038 | WF4 | 10 | 7 | 123-153 | 0.800 | 0.768 | 0.846 | 4.983 |
|  | PlatIS08039 | WF5 | 10 | 6 | 131-187 | 0.600 | 0.579 | 0.700 | 4.026 |
|  | PlatIS08043 | EF1 | 10 | 8 | 131-171 | 0.800 | 0.789 | 0.861 | 5.553 |
|  | PlatIS08048 | EF2 | 9 | 7 | 129-165 | 0.889 | 0.745 | 0.934 | 5.118 |
|  | PlatIS08049 | EF3 | 10 | 7 | 131-167 | 0.600 | 0.763 | 0.294 | 4.851 |
|  | PlatIS08041 | CF2 | 6 | 3 | 133-143 | 0.167 | 0.682 | 0.022 | 3.000 |
|  | PlatIS08042 | CF3 | 5 | 5 | 131-159 | 0.800 | 0.822 | 0.337 | 5.000 |
|  | PlatIS08046 | CF4 | 10 | 6 | 131-181 | 0.600 | 0.632 | 0.413 | 4.158 |
|  | PlatIS08047 | CF1 | 10 | 4 | 129-137 | 0.300 | 0.437 | 0.135 | 3.026 |
|  | PlatIS08055 | SC1 | 10 | 6 | 129-169 | 0.900 | 0.795 | 1.000 | 4.877 |
| **GT-II33** | PlatIS08062 | LPB | 10 | 2 | 177-179 | 0.200 | 0.442 | 0.132 | 1.995 |
|  | PlatIS08011 | NET | 10 | 2 | 177-179 | 0.100 | 0.100 | 1.000 | 1.500 |
|  | PlatIS08012 | CAM | 10 | 2 | 177-179 | 0.300 | 0.395 | 0.479 | 1.984 |
|  | PlatIS08015 | LA1 | 10 | 2 | 177-179 | 0.300 | 0.268 | 1.000 | 1.895 |
|  | PlatIS08021 | LA2 | 10 | 3 | 165-179 | 0.200 | 0.195 | 1.000 | 2.000 |
|  | PlatIS08024 | MIS | 10 | 4 | 177-195 | 0.600 | 0.658 | 1.000 | 3.263 |
|  | PlatIS08030 | WF1 | 10 | 5 | 177-191 | 0.600 | 0.700 | 0.290 | 3.913 |
|  | PlatIS08031 | WF2 | 8 | 7 | 175-189 | 0.750 | 0.850 | 0.291 | 5.615 |
|  | PlatIS08038 | WF4 | 10 | 6 | 175-189 | 1.000 | 0.842 | 0.763 | 5.197 |
|  | PlatIS08039 | WF5 | 10 | 4 | 175-189 | 0.400 | 0.605 | 0.230 | 3.351 |
|  | PlatIS08043 | EF1 | 10 | 4 | 175-181 | 0.400 | 0.489 | 0.657 | 2.957 |
|  | PlatIS08048 | EF2 | 9 | 3 | 175-179 | 0.556 | 0.647 | 0.763 | 2.930 |
|  | PlatIS08049 | EF3 | 10 | 3 | 175-179 | 0.400 | 0.563 | 0.305 | 2.500 |
|  | PlatIS08041 | CF2 | 6 | 3 | 175-179 | 0.500 | 0.667 | 0.724 | 2.985 |
|  | PlatIS08042 | CF3 | 5 | 2 | 177-179 | 0.200 | 0.467 | 0.332 | 2.000 |
|  | PlatIS08046 | CF4 | 10 | 2 | 177-179 | 0.600 | 0.505 | 1.000 | 2.000 |
|  | PlatIS08047 | CF1 | 10 | 3 | 177-181 | 0.400 | 0.637 | 0.135 | 2.893 |
|  | PlatIS08055 | SC1 | 10 | 3 | 175-179 | 0.800 | 0.616 | 0.738 | 2.763 |
| **GT-I13B** | PlatIS08062 | LPB | 10 | 2 | 248-250 | 0.300 | 0.268 | 1.000 | 1.895 |
|  | PlatIS08011 | NET | 10 | 2 | 248-250 | 0.800 | 0.505 | 0.173 | 2.000 |
|  | PlatIS08012 | CAM | 10 | 2 | 248-250 | 0.400 | 0.526 | 0.564 | 2.000 |
|  | PlatIS08015 | LA1 | 10 | 2 | 248-250 | 0.300 | 0.479 | 0.480 | 1.998 |
|  | PlatIS08021 | LA2 | 10 | 3 | 248-252 | 0.700 | 0.563 | 0.733 | 2.500 |
|  | PlatIS08024 | MIS | 10 | 1 | 248 | - | - | - | 1.000 |
|  | PlatIS08030 | WF1 | 10 | 1 | 248 | - | - | - | 1.000 |
|  | PlatIS08031 | WF2 | 8 | 1 | 248 | - | - | - | 1.000 |
|  | PlatIS08038 | WF4 | 10 | 1 | 248 | - | - | - | 1.000 |
|  | PlatIS08039 | WF5 | 10 | 1 | 248 | - | - | - | 1.000 |
|  | PlatIS08043 | EF1 | 10 | 1 | 248 | - | - | - | 1.000 |
|  | PlatIS08048 | EF2 | 9 | 1 | 248 | - | - | - | 1.000 |
|  | PlatIS08049 | EF3 | 10 | 1 | 248 | - | - | - | 1.000 |
|  | PlatIS08041 | CF2 | 6 | 1 | 248 | - | - | - | 1.000 |
|  | PlatIS08042 | CF3 | 5 | 2 | 248-252 | 0.200 | 0.200 | 1.000 | 2.000 |
|  | PlatIS08046 | CF4 | 10 | 3 | 246-250 | 0.800 | 0.679 | 0.751 | 2.978 |
|  | PlatIS08047 | CF1 | 10 | 3 | 246-250 | 0.300 | 0.595 | 0.056 | 2.762 |
|  | PlatIS08055 | SC1 | 10 | 2 | 248-250 | 0.200 | 0.189 | 1.000 | 1.763 |
| **GA-I5B** | PlatIS08062 | LPB | 10 | 1 | 146 | - | - | - | 1.000 |
|  | PlatIS08011 | NET | 10 | 1 | 146 | - | - | - | 1.000 |
|  | PlatIS08012 | CAM | 10 | 1 | 146 | - | - | - | 1.000 |
|  | PlatIS08015 | LA1 | 10 | 1 | 146 | - | - | - | 1.000 |
|  | PlatIS08021 | LA2 | 10 | 1 | 146 | - | - | - | 1.000 |
|  | PlatIS08024 | MIS | 10 | 1 | 146 | - | - | - | 1.000 |
|  | PlatIS08030 | WF1 | 10 | 2 | 146-148 | 0.400 | 0.337 | 1.000 | 1.957 |
|  | PlatIS08031 | WF2 | 8 | 4 | 142-148 | 0.750 | 0.642 | 0.689 | 3.492 |
|  | PlatIS08038 | WF4 | 10 | 3 | 142-146 | 0.600 | 0.468 | 1.000 | 2.484 |
|  | PlatIS08039 | WF5 | 10 | 3 | 142-146 | 0.400 | 0.353 | 1.000 | 2.395 |
|  | PlatIS08043 | EF1 | 10 | 2 | 144-146 | 0.300 | 0.268 | 1.000 | 1.895 |
|  | PlatIS08048 | EF2 | 9 | 2 | 142-146 | 0.111 | 0.111 | 1.000 | 1.556 |
|  | PlatIS08049 | EF3 | 10 | 1 | 146 | - | - | - | 1.000 |
|  | PlatIS08041 | CF2 | 6 | 1 | 146 | - | - | - | 1.000 |
|  | PlatIS08042 | CF3 | 5 | 1 | 146 | - | - | - | 1.000 |
|  | PlatIS08046 | CF4 | 10 | 2 | 142-146 | 0.500 | 0.521 | 1.000 | 2.000 |
|  | PlatIS08047 | CF1 | 10 | 1 | 146 | - | - | - | 1.000 |
|  | PlatIS08055 | SC1 | 10 | 2 | 146-148 | 0.200 | 0.442 | 0.133 | 1.995 |
| **GT-I34** | PlatIS08062 | LPB | 10 | 7 | 210-254 | 0.900 | 0.847 | 0.956 | 5.434 |
|  | PlatIS08011 | NET | 10 | 13 | 220-254 | 1.000 | 0.953 | 0.543 | 8.079 |
|  | PlatIS08012 | CAM | 10 | 15 | 214-282 | 1.000 | 0.974 | 1.000 | 8.816 |
|  | PlatIS08015 | LA1 | 10 | 12 | 224-258 | 1.000 | 0.937 | 0.656 | 7.641 |
|  | PlatIS08021 | LA2 | 10 | 14 | 208-272 | 1.000 | 0.963 | 1.000 | 8.447 |
|  | PlatIS08024 | MIS | 10 | 12 | 228-278 | 1.000 | 0.932 | 0.714 | 7.509 |
|  | PlatIS08030 | WF1 | 10 | 10 | 232-278 | 0.900 | 0.895 | 0.943 | 6.598 |
|  | PlatIS08031 | WF2 | 8 | 12 | 230-282 | 1.000 | 0.967 | 1.000 | 8.500 |
|  | PlatIS08038 | WF4 | 10 | 11 | 218-274 | 0.900 | 0.916 | 0.827 | 7.168 |
|  | PlatIS08039 | WF5 | 10 | 13 | 238-280 | 0.900 | 0.953 | 0.495 | 8.079 |
|  | PlatIS08043 | EF1 | 10 | 11 | 230-288 | 0.900 | 0.916 | 0.833 | 7.071 |
|  | PlatIS08048 | EF2 | 9 | 16 | 218-286 | 1.000 | 0.987 | 1.000 | 9.412 |
|  | PlatIS08049 | EF3 | 10 | 15 | 206-274 | 1.000 | 0.958 | 1.000 | 8.483 |
|  | PlatIS08041 | CF2 | 6 | 8 | 216-272 | 0.833 | 0.924 | 0.494 | 7.136 |
|  | PlatIS08042 | CF3 | 5 | 9 | 226-272 | 1.000 | 0.978 | 1.000 | 9.000 |
|  | PlatIS08046 | CF4 | 10 | 15 | 252-288 | 0.800 | 0.968 | 0.066 | 8.684 |
|  | PlatIS08047 | CF1 | 10 | 9 | 240-286 | 0.700 | 0.889 | 0.277 | 6.361 |
|  | PlatIS08055 | SC1 | 10 | 9 | 226-264 | 0.700 | 0.889 | 0.287 | 6.361 |
| **GT-I49** | PlatIS08062 | LPB | 10 | 4 | 138-156 | 0.500 | 0.553 | 0.604 | 3.220 |
|  | PlatIS08011 | NET | 10 | 3 | 138-146 | 0.500 | 0.489 | 0.652 | 2.789 |
|  | PlatIS08012 | CAM | 10 | 3 | 138-146 | 0.600 | 0.484 | 1.000 | 2.720 |
|  | PlatIS08015 | LA1 | 10 | 2 | 138-144 | 0.500 | 0.479 | 1.000 | 1.998 |
|  | PlatIS08021 | LA2 | 10 | 2 | 138-144 | 0.500 | 0.521 | 1.000 | 2.000 |
|  | PlatIS08024 | MIS | 10 | 2 | 144-150 | 0.200 | 0.189 | 1.000 | 1.763 |
|  | PlatIS08030 | WF1 | 10 | 3 | 142-150 | 0.600 | 0.668 | 0.709 | 2.955 |
|  | PlatIS08031 | WF2 | 8 | 5 | 138-150 | 0.750 | 0.725 | 0.429 | 4.339 |
|  | PlatIS08038 | WF4 | 10 | 4 | 138-146 | 0.800 | 0.689 | 1.000 | 3.394 |
|  | PlatIS08039 | WF5 | 10 | 5 | 138-154 | 0.800 | 0.695 | 1.000 | 3.762 |
|  | PlatIS08043 | EF1 | 10 | 7 | 138-150 | 0.800 | 0.847 | 0.362 | 5.319 |
|  | PlatIS08048 | EF2 | 9 | 3 | 138-144 | 0.333 | 0.464 | 0.247 | 2.748 |
|  | PlatIS08049 | EF3 | 10 | 5 | 138-158 | 0.500 | 0.568 | 0.270 | 3.658 |
|  | PlatIS08041 | CF2 | 6 | 2 | 144-152 | 0.333 | 0.545 | 0.479 | 2.000 |
|  | PlatIS08042 | CF3 | 5 | 5 | 138-150 | 0.800 | 0.844 | 0.847 | 5.000 |
|  | PlatIS08046 | CF4 | 10 | 1 | 138 | - | - | - | 1.000 |
|  | PlatIS08047 | CF1 | 10 | 3 | 138-148 | 0.300 | 0.416 | 0.477 | 2.457 |
|  | PlatIS08055 | SC1 | 10 | 1 | 144 | - | - | - | 1.000 |

Supplementary Table 4: Summary statistics of sample size and microsatellite diversity for the analyzed populations (mean values across 13 microsatellite loci; see supplement for locus-specific information).

| **Population** | **N** | **No. of alleles** | **Allelic richness** | **H_0_** | **H_E_** |
| --- | --- | --- | --- | --- | --- |
| LPB | 10 | 4.923 | 3.787 | 0.569 | 0.589 |
| NET | 10 | 4.154 | 3.170 | 0.477 | 0.452 |
| CAM | 10 | 4.538 | 3.335 | 0.471 | 0.482 |
| LA1 | 10 | 4.385 | 3.274 | 0.523 | 0.506 |
| LA2 | 10 | 4.692 | 3.520 | 0.598 | 0.543 |
| MIS | 10 | 3.846 | 2.950 | 0.400 | 0.416 |
| WF1 | 10 | 4.000 | 3.147 | 0.492 | 0.523 |
| WF2 | 8 | 5.615 | 4.547 | 0.692 | 0.685 |
| WF4 | 10 | 6.077 | 4.445 | 0.708 | 0.662 |
| WF5 | 10 | 6.462 | 4.619 | 0.677 | 0.673 |
| EF1 | 10 | 5.538 | 4.163 | 0.608 | 0.611 |
| EF2 | 9 | 6.538 | 4.647 | 0.607 | 0.610 |
| EF3 | 10 | 6.231 | 4.210 | 0.523 | 0.551 |
| CF2 | 6 | 3.308 | 3.119 | 0.410 | 0.479 |
| CF3 | 5 | 4.154 | 4.154 | 0.508 | 0.615 |
| CF4 | 10 | 4.692 | 3.464 | 0.538 | 0.545 |
| CF1 | 10 | 4.308 | 3.245 | 0.400 | 0.496 |
| SC1 | 10 | 4.000 | 3.218 | 0.477 | 0.475 |

Supplementary Table 5: Summary statistics of sample size and life-history traits for male sailfin

mollies (*Poecilia latipinna*) from 14 populations across their geographical distribution. GSI: gonadosomatic index.

| **Genetic Cluster** | **Population** | **N** | **SL [mm]** | **Lean Mass* [mg]** | **Fat Content [%]** | **GSI* [%]** |
| --- | --- | --- | --- | --- | --- | --- |
| 1 | NET | 18 | 33.50 ± 4.95 | 181.87 ± 33.35 | 4.77 ± 3.85 | 1.07 ± 0.30 |
|  | CAM | 15 | 31.33 ± 6.62 | 199.36 ± 33.42 | 5.82 ± 4.76 | 1.20 ± 0.31 |
|  | LA1 | 9 | 27.56 ± 4.00 | 209.71 ± 34.05 | 2.22 ± 3.98 | 1.42 ± 0.30 |
|  | LA2 | 11 | 29.73 ± 4.17 | 180.49 ± 34.53 | 3.27 ± 2.31 | 0.95 ± 0.30 |
|  | MIS | 10 | 37.10 ± 9.05 | 203.12 ± 33.87 | 2.16 ± 2.51 | 1.40 ± 0.28 |
|  | LPB | 10 | 29.90 ± 2.42 | 180.91 ± 33.58 | 7.77 ± 4.44 | 1.20 ± 0.28 |
|  | Mean |  | 31.75 ± 6.13 | 190.96 ± 35.81 | 4.00 ± 4.02 | 1.19 ± 0.31 |
| 2 | WF1 | 14 | 33.07 ± 4.05 | 167.39 ± 35.61 | 9.77 ± 5.47 | 1.07 ± 0.31 |
| 3 | WF5 | 10 | 28.00 ± 5.54 | 200.44 ± 36.18 | 6.41 ± 5.99 | 1.32 ± 0.32 |
| 4 | CF1 | 16 | 39.75 ± 6.61 | 144.98 ± 35.52 | 8.35 ± 5.22 | 1.26 ± 0.32 |
|  | CF3 | 12 | 34.17 ± 7.27 | 167.17 ± 33.39 | 0.70 ± 1.54 | 0.93 ± 0.28 |
|  | EF2 | 20 | 37.85 ± 3.41 | 188.39 ± 34.79 | 4.18 ± 3.92 | 0.98 ± 0.31 |
|  | EF3 | 15 | 30.60 ± 5.44 | 203.35 ± 33.54 | 6.65 ± 3.17 | 1.14 ± 0.31 |
|  | Mean |  | 35.90 ± 6.53 | 178.38 ± 37.11 | 5.25 ± 4.84 | 1.08 ± 0.33 |
| 5 | CF4 | 20 | 31.60 ± 6.65 | 194.71 ± 35.67 | 2.63 ± 1.58 | 1.07 ± 0.31 |
| 6 | SC1 | 15 | 29.47 ± 5.04 | 199.56 ± 36.02 | 7.77 ± 4.44 | 1.50 ± 0.32 |

* estimated marginal means from general linear models with SL as a covariate (at SL = 32.81 mm).

Supplementary Table 6: Summary statistics of sample size and life-history traits for female sailfin mollies (*Poecilia latipinna*) from 11 populations across their geographical distribution. RA: reproductive allocation.

| **Genetic Cluster** | **Population** | **N** | **SL [mm]** | **Lean Mass* [mg]** | **Fat Content+ [%]** | **Fecundity*** | **Embryo Lean Mass+ [mg]** | **Embryo Fat Content+ [%]** | **RA+ [%]** |
| --- | --- | --- | --- | --- | --- | --- | --- | --- | --- |
| 1 | CAM | 17 | 35.12 ± 3.14 | 343.76 ± 34.63 | 10.09 ± 3.25 | 23.62 ± 8.19 | 3.11 ± 0.61 | 14.41 ± 3.53 | 18.49 ± 5.01 |
|  | LA1 | 16 | 39.13 ± 3.03 | 316.40 ± 30.72 | 1.34 ± 2.88 | 32.01 ± 7.27 | 2.72 ± 0.54 | 12.57 ± 3.12 | 23.27 ± 4.43 |
|  | MIS | 16 | 40.94 ± 2.08 | 316.24 ± 30.48 | 1.79 ± 2.83 | 16.98 ± 7.21 | 3.70 ± 0.53 | 15.66 ± 3.07 | 18.72 ± 4.36 |
|  | LPB | 7 | 47.43 ± 5.88 | 341.27 ± 33.39 | 7.14 ± 3.14 | 28.16 ± 7.90 | 3.51 ± 0.59 | 17.25 ± 3.40 | 23.14 ± 4.83 |
|  | Mean |  | 39.46 ± 4.99 | 329.42 ± 32.57 | 5.09 ± 3.05 | 25.19 ± 7.71 | 3.26 ± 0.57 | 14.97 ± 3.31 | 20.90 ± 4.70 |
| 2 | WF1 | 16 | 41.31 ± 1.66 | 291.89 ± 30.55 | 9.33 ± 2.96 | 19.27 ± 7.23 | 3.89 ± 0.56 | 16.56 ± 3.21 | 22.04 ± 4.55 |
| 3 | WF5 | 14 | 38.50 ± 3.06 | 295.91 ± 30.95 | 6.39 ± 2.87 | 20.60 ± 7.32 | 3.60 ± 0.54 | 17.84 ± 3.11 | 21.84 ± 4.42 |
| 4 | CF1 | 15 | 45.80 ± 3.14 | 246.24 ± 34.10 | 10.40 ± 3.16 | 26.00 ± 8.07 | 3.77 ± 0.60 | 18.05 ± 3.43 | 28.03 ± 4.88 |
|  | CF3 | 11 | 37.82 ± 3.31 | 299.19 ± 31.16 | 3.02 ± 2.91 | 14.28 ± 7.37 | 4.94 ± 0.55 | 12.73 ± 3.15 | 19.23 ± 4.47 |
|  | EF3 | 12 | 39.25 ± 4.83 | 379.12 ± 30.62 | 7.60 ± 2.85 | 27.94 ± 7.24 | 2.60 ± 0.54 | 17.04 ± 3.10 | 16.70 ± 4.39 |
|  | Mean |  | 41.42 ± 5.18 | 308.18 ± 30.79 | 7.01 ± 2.85 | 22.74 ± 7.29 | 3.77 ± 0.54 | 15.94 ± 3.09 | 21.32 ± 4.39 |
| 5 | CF4 | 14 | 43.07 ± 5.77 | 321.99 ± 31.29 | 3.08 ± 2.90 | 10.45 ± 7.40 | 4.47 ± 0.55 | 17.81 ± 3.14 | 16.37 ± 4.46 |

* estimated marginal means from general linear models with SL as a covariate (at SL = 40.49 mm).

^+^ estimated marginal means from general linear models with SL and embryonic stage of development as covariates (at SL = 40.49 mm, Stage = 26.76).

|  | **Component (males)** | | **Component (females)** | |
| --- | --- | --- | --- | --- |
|  | **1** | **2** | **1** | **2** |
| Water Temperature [ºC] | **0.879** | 0.147 | **0.894** | 0.145 |
| pH | 0.313 | **0.764** | 0.518 | 0.520 |
| Turbidity | -0.172 | **0.844** | 0.128 | **0.894** |
| DO [%] | **0.820** | -0.404 | **0.768** | -0.499 |
| Salinity [ppt] | **0.726** | 0.149 | **0.826** | -0.158 |

Supplementary Table 7: Axis loadings for sex-specific Principal Component Analysis of environmental variables for each sample site. Variables with axis loadings >|600| are in bold.


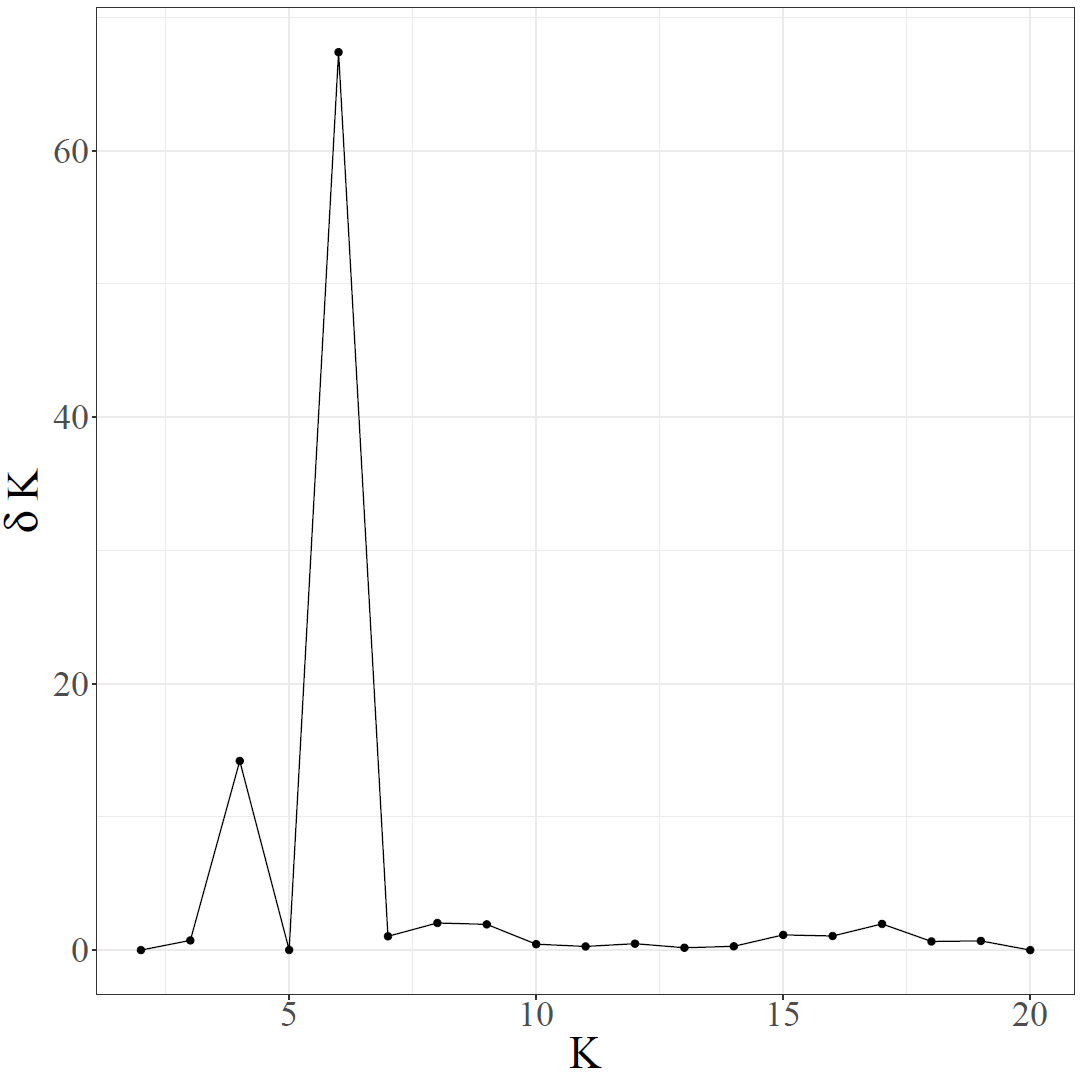


Supplementary Figure 1: Evanno-plot illustrating the relationship between the number of clusters (K) and the rate of change in the log-likelihood of the STRUCTURE output data as indicated by ΔK. The optimal number of genetic clusters is determined by identifying the peak value of δK.


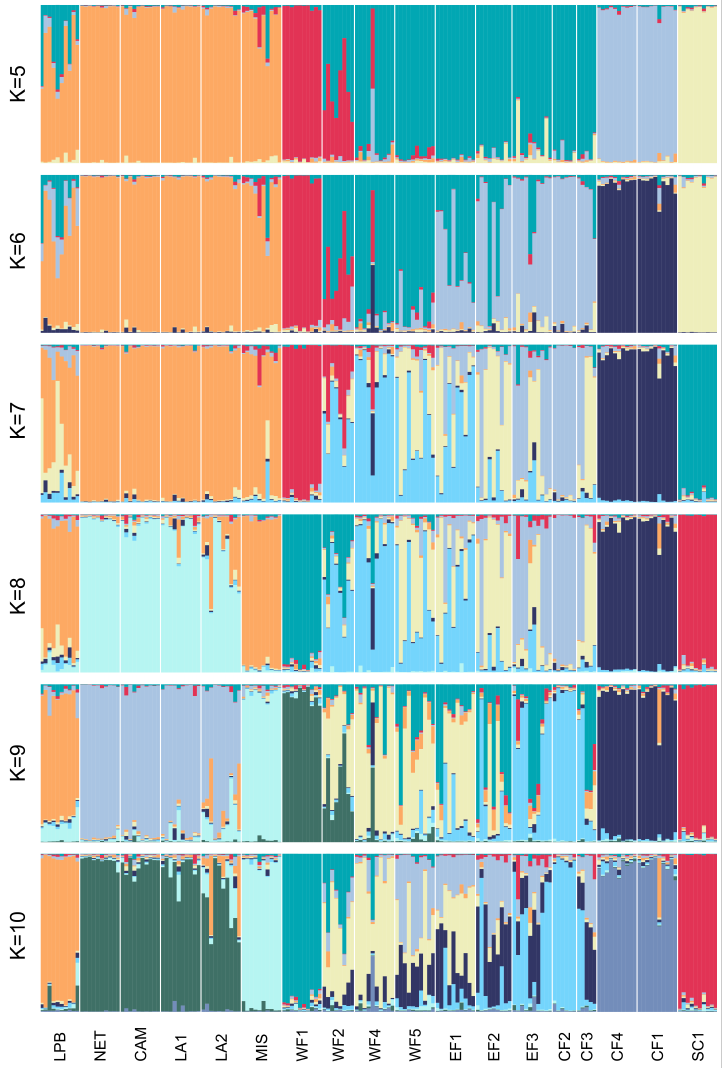


Supplementary Figure 2: Results of complementary STRUCTURE analyses (K=5−10). Each bar represents an individual genotype with single relative membership probability illustrated by distinct colors. Individual genotypes are ordered by their respective sampling location.


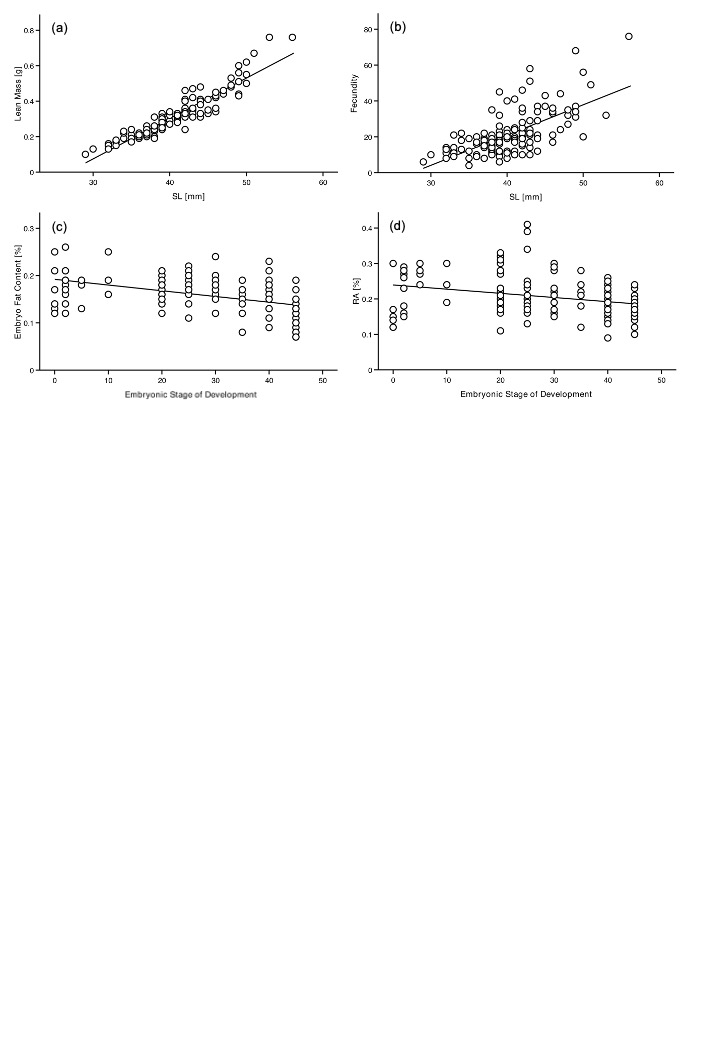


Supplementary Figure 3: Visualization of the significant effects of the covariates SL and Embryonic Stage of Development on female life-history traits. (a) Lean mass, (b) fecundity, (c) embryo fat content and (d) reproductive allocation (RA).

References

1. Greven H: **Gonads, genitals, and reproductive biology**. In: *Ecology and Evolution of Poeciliid Fishes.* Edited by Evans JP, Pilastro A, Schlupp I. Chicago: University of Chicago Press; 2011.

2. Girndt A, Riesch R, Schroeder C, Sehlupp I, Plath M, Tiedemann R: **Multiple paternity in different populations of the sailfin molly, *Poecilia latipinna***. *Animal Biology* 2012, **62**(3):245-262.

3. Travis J, Trexler JC, Mulvey M: **Multiple Paternity and Its Correlates In Female *Poecilia latipinna* (Poeciliidae)**. *Copeia* 1990(3):722-729.

4. Trexler JC, Travis J, Dinep A: **Variation among populations of the sailfin molly in the rate of concurrent multiple paternity and its implications for mating-system evolution**. *Behavioral Ecology and Sociobiology* 1997, **40**(5):297-305.

5. Trexler JC, Travis J: **Phenotypic Plasticity in the Sailfin Molly, *Poecilia latipinna* (Pisces: Poeciliidae). I. Field Experiments**. *Evolution* 1990, **44**(1):143-156.

6. Schlupp I, McKnab R, Ryan MJ: **Sexual harassment as a cost for molly females: Bigger males cost less**. *Behaviour* 2001, **138**:277-286.

7. Travis J, Woodward BD: **Social Context and Courtship Flexibility in Male Sailfin Mollies *Poecilia latipinna* Pisces Poeciliidae**. *Animal Behaviour* 1989, **38**(6):1001-1011.

8. Page LM, Burr BM: **Peterson field guide to freshwater fishes of North America north of Mexico**: Houghton Mifflin Harcourt; 2011.

9. Snelson FF, Jr.: **Indeterminate growth in males of the sailfin molly, *Poecilia latipinna***. *SO - Copeia 1982(2) 1982 296-304* 1982.

10. Hubbs C, Edwards RJ, Garrett GP: **An Annotated Checklist of the Freshwater Fishes of Texas with Keys to Identification of Species**. *Texas Journal of Science* 1991, **43**(4 SUPPL):1-56.

11. Snelson FF, Jr.: **Size and Morphological Variation in Males of the Sailfin Molly *Poecilia latipinna***. *Environmental Biology of Fishes* 1985, **13**(1):35-48.

12. Palacios M, Voelker G, Rodriguez LA, Mateos M, Tobler M: **Phylogenetic analyses of the subgenus *Mollienesia* (*Poecilia*, Poeciliidae, Teleostei) reveal taxonomic inconsistencies, cryptic biodiversity, and spatio-temporal aspects of diversification in Middle America**. *Molecular Phylogenetics and Evolution* 2016, **103**:230-244.

13. Meredith RW, Pires MN, Reznick DN, Springer MS: **Molecular phylogenetic relationships and the evolution of the placenta in *Poecilia* (*Micropoecilia*)(Poeciliidae: Cyprinodontiformes)**. *Molecular Phylogenetics and Evolution* 2010, **55**(2):631-639.

14. Schartl M, Wilde B, Schlupp I, Parzefall J: **Evolutionary origin of a parthenoform, the Amazon molly *Poecilia formosa*, on the basis of a molecular genealogy**. *Evolution* 1995, **49**(5):827-835.

15. Schlupp I: **The evolutionary ecology of gynogenesis**. *Annual Review of Ecology Evolution and Systematics* 2005, **36**:399-417.

16. Farr JATJ: **Fertility Advertisement by Female Sailfin Mollies *Poecilia latipinna* Pisces Poeciliidae**. *Copeia* 1986, **1986**(2):467-472.

17. Fraser BA, Janowitz I, Thairu M, Travis J, Hughes KA: **Phenotypic and genomic plasticity of alternative male reproductive tactics in sailfin mollies**. *Proceedings of the Royal Society B-Biological Sciences* 2014, **281**(1781).

18. Parzefall J: **Zur vergleichenden Ethologie verschiedener *Mollienesia* Arten einschliesslich einer Höhlenform von *M. sphenops*.** *Behaviour* 1969, **33**:1-37.

19. Ptacek MB, Travis J: **Mate choice in the sailfin molly, *Poecilia latipinna***. *Evolution* 1997, **51**(4):1217-1231.

20. Trexler JC: **Resource availability and plasticity in offspring provisioning: Embryo nourishment in sailfin mollies**. *Ecology* 1997, **78**(5):1370-1381.

21. Pirroni S, Meka J, Jourdan J, Santi F, Plath M, Langerhans RB, Riesch R: **Variability in maternal provisioning and offspring quality in eastern mosquitofish (*Gambusia holbrooki*).** *Aqua, International Journal of Ichthyology* 2022, **28**(2):79 - 95.

22. Trexler JC: **Variation in the Degree of Viviparity in the Sailfin Molly, *Poecilia latipinna***. *Copeia* 1985, **1985**(4):999-1004.

23. Riesch R, Schlupp I, Langerhans RB, Plath M: **Shared and Unique Patterns of Embryo Development in Extremophile Poeciliids**. *PLoS ONE* 2011, **6**(11):1-11.
